# Application of Statistical Shape Models to Standard Lumbar MRI for Stenosis Treatment Stratification: Severe Versus Normal Stenosis

**DOI:** 10.1002/jsp2.70189

**Published:** 2026-06-07

**Authors:** Mary H. Foltz, Alexandra Seidenstein, Wesley M. Durand, Amit Jain, Jill M. Middendorf

**Affiliations:** ^1^ Department of Mechanical Engineering Johns Hopkins University Baltimore Maryland USA; ^2^ Department of Orthopaedic Surgery Johns Hopkins University Baltimore Maryland USA

**Keywords:** axial MRI, low back pain, lumbar spinal stenosis, machine learning, morphological analysis, principal component analysis, quantitative imaging, statistical shape modeling, surgical planning

## Abstract

**Background:**

Lumbar spinal stenosis is a prevalent and debilitating diagnosis, which in severe cases requires surgical treatment to relieve nerve root pressure. Often, treatment plans are based in part on subjective, qualitative, and limited MRI assessment. Statistical shape models (SSMs) have the potential to improve treatment indications by uncovering morphological features that work synergistically but are difficult to assess independently or directly quantify. This study examined whether 2D SSM using standard clinically relevant MRIs can differentiate between severe and normal stenosis patients in a population with low back pain.

**Methods:**

A total of 62 patients were analyzed from an open‐access Lumbar Spine MRI dataset, with variable parameters, and classified as severe or normal stenosis (at L45). Intervertebral disc (IVD) and posterior element (PE) edges were extracted using the SegNet algorithm, then aligned via generalized Procrustes analysis. Aligned shapes were used for principal component analysis and principal components (PCs) were evaluated for IVD and PE independently and together. To observe if SSMs improved from anatomical measurements, facet angles, spinous process length, IVD, and thecal sac diameters were manually extracted. ANOVA and ROC analysis was run to determine the measurements' ability to discriminate between groups.

**Results:**

PC1 explained 38% and 32% of the IVD and combined (IVD & PE) shape variances and had a clear difference between the severe and normal stenosis groups (*p* < 0.01) with moderate to strong discriminatory power (AUC = 0.89, 0.83). In comparison to anatomical measurements, the combined SSM's ability to distinguish between groups was comparable to the thecal sac diameter (AUC = 0.84) and exceeded all other traditional anatomical measures.

**Conclusions:**

SSMs were able to distinguish between the severe and normal stenosis groups, specifically PC1 for both IVD and combined SSMs. Hence, this study demonstrates that SSMs could be a quantitative tool to improve stenosis diagnosis and treatment planning using clinical 2D MRIs.

## Graphical abstract

Statistical shape models (SSMs) derived from standard 2D lumbar MRIs capture coordinated morphological features that distinguish severe from normal spinal stenosis. Both intervertebral disc and combined models provide strong discriminatory power, comparable to or exceeding traditional anatomical measurements. These findings support SSMs as a quantitative, objective tool to enhance stenosis diagnosis and inform clinical decision‐making.

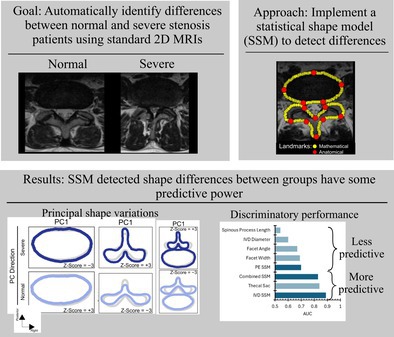

**Abbreviations**
AP: anterior posterior
AUC: area under the curve
CI: confidence interval
CT: computed tomography
GLM: generalized linear model
GPA: Generalized Procrustes Analysis
ICCs: intraclass correlation coefficients
IoU: intersection over union
IVD: intervertebral disc
ML: medial lateral
MRI: magnetic resonance imaging
PC: principal component
PCA: principal component analysis
PE: posterior elements
RMS: root mean squared
ROC: receiver operating characteristic
SSM: statistical shape model

## 1 Introduction

Lumbar spinal stenosis has become increasingly prevalent, contributing to more than 500 000 spinal decompression surgeries annually in the United States alone [1, 2]. However, the surgical success rate, meaning the ability to reduce clinical symptoms of severe stenosis, remains suboptimal, with reported effectiveness as low as 40% [3]. Some of the ineffectiveness of these surgeries comes from the diagnosis and surgical planning process. Once conservative treatment of clinical symptoms fails, clinicians often rely on qualitative assessments of magnetic resonance images (MRI) to decide the next treatment strategy—often surgical intervention. These qualitative MRI assessments often result in subjective interpretations and limited reproducibility [4]. Thus, there is a need for a quantitative consistent approach to identify which patients are most likely to benefit from surgical intervention to reduce variability in surgical outcomes and improve patient care.

Statistical shape modeling (SSM) presents as a promising approach to improve surgical planning. As a form of unsupervised machine learning algorithm, SSM uses principal component analysis (PCA) to capture population‐level variations in anatomical shapes. SSMs can uncover combinations of morphological features (e.g., simultaneous narrowing of the thecal sac and facet joint hypertrophy) that may act synergistically but are difficult to assess independently or directly quantify. Unlike traditional measurements, SSMs provide a compact, interpretable set of “shape modes” that reflect the principal axes of anatomical variation, including subtle deformations that may not be visually obvious but carry clinical importance [5]. Typically, SSMs of the lumbar spine have been developed using 3D reconstructions of the vertebral bodies collected from CT scans [6, 7]. These SSMs have detected variations in spinal curvature and vertebral body sizes due to age, sex, posture, and spondylolisthesis [7, 8, 9, 10, 11], but CT scans are not standard clinical practice for stenosis treatment. A few studies have used SSMs to classify 2D spinal geometry including the ability to detect lumbar intervertebral disc (IVD) degenerative changes associated with the sagittal lumbar spinal shape [12, 13, 14, 15]. However, these 2D SSMs have never linked vertebral body and IVD shapes directly to distinguish between severe stenosis (that may require further surgical evaluation) from normal stenosis (that occurs due, in part, to natural degeneration, aging, or both) to use and inform on treatment.

Lumbar spinal stenosis is associated with a range of key features on axial MRIs, specifically, providing direct visualization of foraminal compression, facet joint morphology, and a decrease in thecal sac's anterior–posterior (AP) diameter [16], offering greater specificity than sagittal views for assessing stenosis severity. The thecal sac is considered important due to the perceived compression of the nerve root, and the expectation that the decompression surgery will reduce compression of the nerve root and therefore reduce pain. In addition, individuals diagnosed with severe stenosis also commonly experience IVD bulging and/or herniations, which can be observed via MRI as an increase in the IVD diameters, AP and medial‐lateral (ML) [3, 17]. Furthermore, on the posterior side of the spine, the facets trend towards sagittal alignment (i.e., parallel to the spinous process) in patients diagnosed with severe stenosis [18, 19, 20]. These differences not only indicate a need for surgery but may point to a biomechanical mechanism for the onset of degeneration and potential for surgical success [21]. However, these anatomical measurements of the thecal sac and IVD do not account for the natural changes in size due, in part, to biological sex, age, and genetic predispositions [7, 22, 23, 24]. Additionally, these measures are not routinely integrated into clinical workflows due to the time‐consuming nature of manual acquisition and moderate reproducibility across observers [16]. Therefore, a method is needed to quantify the types of complex anatomical changes that are currently evaluated subjectively on axial MRIs. Specifically, an approach that merges the objectivity of quantitative SSMs with the comprehensiveness of qualitative assessment and the potential for automation is desired.

Therefore, as a proof‐of‐concept, this study used a pre‐existing dataset of patients with low back pain, grouped by a spinal expert, to explore whether a 2D SSM could distinguish severe and normal stenosis features on axial MRIs. Since the L45 IVD level is the most commonly affected site for severe stenosis, the L45 axial MRI slice was chosen for this analysis [25]. Unlike many previous SSMs, which use a 3D analysis or specialized MRI and CT imaging of patients, this study chose to apply SSMs to current clinical imaging modalities (average resolution 2D MRI) that would not require modifications to the current patient imaging process. Furthermore, while sagittal MRIs are valuable for assessing overall spinal alignment and identifying the general spinal level of stenosis, axial MRIs provide more critical anatomical features such as canal narrowing, lateral recess and foraminal compression, facet hypertrophy, and side‐specific pathology. This axial perspective and the use of a single image could provide balance between clinical decision‐making assistance and computational efficiency. As such, this study was designed as a proof‐of‐concept to evaluate whether shape‐based modeling of a single, clinically routine axial MRI slice could differentiate severe stenosis development from normal degeneration, aging, or both in a cohort of individuals with low back pain, establishing an approach with potential to support clinical decision‐making.

## 2 Materials and Methods

This study implemented a retrospective analysis based on an open‐source lumbar spinal MRI dataset of individuals with symptomatic back pain from a single institution using clinical scanners and protocols, with varying imaging parameters across patients [26, 27]. This study focused on the axial MRIs, as these sequences provided the most consistent visualization of central and lateral (foraminal) canal structures relevant to stenosis. An orthopedic surgeon (AJ) was blinded to clinical information and radiologic reports, then reviewed MRIs and assigned each patient into either the severe or normal stenosis group. The severe stenosis group was determined solely from MRI‐based interpretation based on MRI features consistent with patients that would typically warrant surgical evaluation by an orthopedic surgeon. These metrics included all features described in the Lee and Schiza's grading systems such as high degree of dural sac compression, reduced cerebrospinal fluid space, and nerve root impingement. To improve consistency in classification, all MRIs originally reviewed by the spine surgeon were independently re‐evaluated by a spine clinical fellow using the Lee and Schizas classification systems, which use the same metrics. This analysis was performed to assess the feasibility of an imaging‐based SSM approach with the intention that this process could assist in the clinical decision‐making process.

### 2.1 Statistical Shape Models (SSMs)

#### 2.1.1 Selection Criteria

MRIs were assigned to the severe stenosis group if the clinician would recommend spinal decompression surgery at the L45 level based on the severity of lumbar spinal stenosis or a combination of stenosis and IVD impingement. Of note, clinical symptoms were unknown and assumed to be consistent with those of severe stenosis; ultimately, images raised sufficient concern to warrant further consideration for surgical evaluation depending on clinical symptoms. MRIs from patients under 50 years of age, those with prior spinal surgery, spinal fusion recommendations, or other spinal pathologies such as cysts, metastatic disease, spondylolisthesis, or scoliosis were excluded. Patients whose imaging did not suggest the need for surgical intervention were assigned to the normal stenosis group (stenosis typical due to normal degeneration, aging, or both).

Following clinical examination, 15 images were removed due to poor image quality (blurry), and 63 images were removed because the patients were indicated for a different surgery, had a prior spinal surgery, or had a spinal deformity. To avoid any skews in the primary shapes that might be caused by having a larger normal than severe stenosis group, the Mersenne Twister algorithm for SSM training was used to randomly select normal stenosis patients for the training and test datasets and all severe stenosis patients were used in the training dataset [28]. The trained SSMs were tested on an independent subset of normal stenosis MRIs, demonstrating consistent results between the training and test groups. While a k‐fold cross‐validation was run on the training dataset to determine the model's robustness (see Methods: Principal Component Analysis [PCA]). After running the PCA and identifying the principal shapes, the normal test group was analyzed using the principal shapes from the SSM.

#### 2.1.2 Pre‐Process Data

**FIGURE 1:**
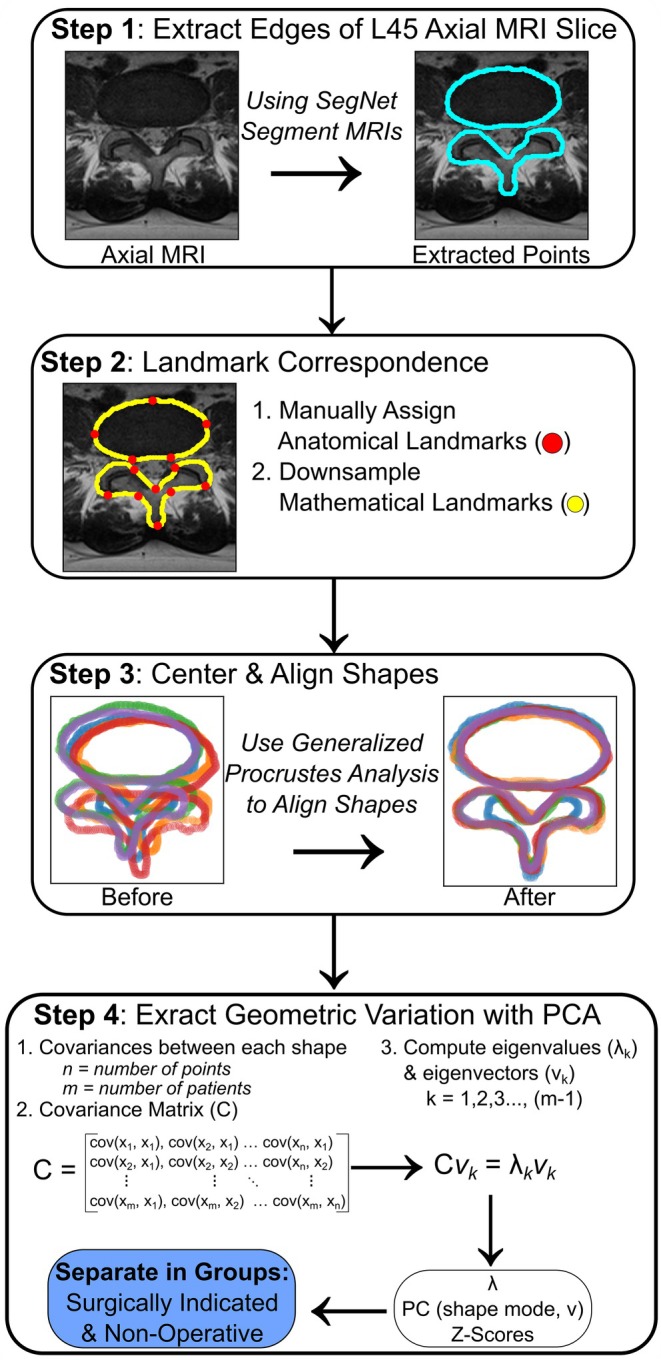
Overview of the statistical shape modeling workflow. (Step 1) Axial MRIs were segmented using a deep learning‐based SegNet model to identify the intervertebral disc and posterior elements. (Step 2) Edge contours were extracted and resampled between manually identified anatomical landmarks to generate consistent point sets. (Step 3) Shapes are aligned across patients using generalized Procrustes analysis to remove the effects of translation, rotation, and scale. (Step 4) Principal component analysis (PCA) was then applied to the aligned shapes to identify dominant modes of variation and build the statistical shape model, extracting the eigenvalues, shape modes, and z‐scores then breaking the results into their respective group (severe and normal stenosis).

Axial MRIs were chosen to create SSMs because the axial views provide bony surface and soft tissue information (facet orientation, foraminal spacing, thecal sac diameter, IVD diameters), which relate to severe stenosis. From a single axial image, the IVD and posterior elements (PE) were segmented (Figure 1, step 1) using the SegNet‐TL80 architecture—a convolutional neural network‐based segmentation algorithm previously developed and validated for lumbar spine segmentation by *Al‐Kafri* et al. [27]. The SegNet‐TL80 model employs transfer learning with an 80:20 training‐to‐testing data split and was trained on the same dataset in the present study. This model demonstrated high segmentation accuracy for both the IVD and PE regions, with class accuracies of 0.99 and 0.96, and intersection‐over‐union (IoU) values of 0.92 and 0.78, respectively. Boundary delineation quality was further confirmed using semantic contour‐based scores, with IVD/PE scores of 0.21/0.19 (tolerance = 1‐pixel), 0.60/0.54 (tolerance = 2‐pixels), and 0.81/0.72 (tolerance = 3‐pixels). These metrics indicated a robust segmentation performance suitable for downstream shape analysis. Hence, using the SegNet‐TL80 model, points along the edge of the IVD and PE regions were automatically extracted from each image. To ensure anatomical accuracy, all segmentations were visually inspected by an experienced spine MRI reviewer; any images with incomplete or inaccurate region delineation were excluded prior to shape alignment and PCA.

Prior to running the SSM, pre‐processing of the segmented regions was required to ensure each shape could be directly and accurately compared. The first step was landmark correspondence (Figure 1, step 2), which refers to a process to ensure that each point within an image corresponds to the same anatomical location on all images. During landmark correspondence, 13 key anatomical landmarks were manually identified and selected. To assess reproducibility, a second independent rater repeated the landmarking process, and the original rater performed a second round of labeling. Inter‐ and intra‐rater reliability were quantified using two‐way mixed‐effects intraclass correlation coefficients (ICCs), and root‐mean‐squared (RMS) Euclidean distances between corresponding landmarks were reported. These anatomical landmarks were chosen as clearly defined regions of the spine, such as the posterior most point in the spinous process and the anterior most point on the IVD. Then, mathematical landmarks were created by evenly redistributing points between the anatomical landmarks such that a total of 559 mathematical landmarks were used. This process ensured that the subsequent SSMs were built on a consistent and comparable set of data points across all patients.

The second pre‐processing step was shape alignment (Figure 1, step 3), which aligned all the shapes to a common reference frame to ensure that the SSM captured shape variation rather than patient position, patient size, or orientation artifacts. The shape alignment process was completed using a previously established generalized Procrustes analysis (GPA), which involves translating, rotating, scaling, and centering the shapes from the training set [29]. First, to remove the effect of position, each shape was translated so that its centroid was at its origin. To remove scale differences, each shape was scaled by dividing by the image size defined as the square root of the sum of the squared distances from each landmark to the centroid. To remove rotation effects, all shapes were iteratively rotated to minimize the Procrustes distance to a reference shape. An initial reference shape was defined as the mean shape, and the process was repeated until the mean shape converged. This alignment process ensured that the subsequent SSM reflected true shape variation rather than differences due to alignment or size. Following shape alignment, the data was flattened and all landmarks for all images were placed into a single matrix. Each row of the matrix corresponded to a single image, while each column of the matrix corresponded to a given *x* or *y* component of a known landmark.

#### 2.1.3 Principal Component Analysis (PCA)

The main feature of SSMs is the use of PCA to understand the shape variations that can explain differences within a given population. PCA was applied to the aligned shapes (training image set), then the eigenvalues (variance explained by each shape mode, $\lambda_{i}$) and eigenvectors (principal component [PC] or shape mode) that characterize the primary modes of shape variation (Figure 1, step 4) were extracted [30]. Explained variance was calculated as a proportion of the total variance explained by each PC (i.e., shape mode).

$$\text{Explained Variance for}\;{\mathrm{PC}}_{i}=\frac{\lambda_{i}}{\sum_{j}\lambda_{j}}\times 100\%$$

where i was the shape mode or PC number and j was the total number of PCs. Eigenvalues identified the contribution of each PC (shape mode) to the total variation and were ordered from largest variance (i.e., PC1) to smallest. Z‐scores informed how different a patient's shape was from the mean shape along each PC in standard deviations. Shape reconstructions used the z‐scores with the PC to rebuild or create a shape. The number of PCs retained for further analysis was determined by excluding PCs beyond 80% of the cumulative explained variance and individual PCs that account for < 5% of the total variance, since low‐variance components are more likely to reflect non‐systematic variations rather than meaningful anatomical differences. Principal components that when plotted looked noisy as well as any PCs that made the variance explained go above 80% were omitted from post SSM group analysis [30]. To reduce overfitting, GPA and PCA were performed using only the training dataset, then the resulting mean shape and eigenvectors were applied to the independent test dataset for evaluation.

SSM validation was assessed using three standard metrics: compactness, generalization, and specificity. Compactness was evaluated using cumulative explained variance as a function of the retained PCs, with boundaries described in the prior paragraph. Generalization was quantified by using reconstruction error (root mean squared [RMS] Euclidean distance and normalized to the number of landmark points) and cross‐validation by projecting left‐out shapes onto the PCA space and computing the RMS Euclidean distance between reconstructed and aligned shapes. Furthermore, the inter‐patient anatomical variability was quantified as the mean squared distance of aligned shapes from the mean shape to interpret the generalization. Specificity was assessed by sampling random shapes (*n* = 500 samples) from the PCA space and measuring the distance to the nearest real training shape, using RMS Euclidean distance and normalized to the number of landmark points, with lower errors indicating higher anatomical plausibility.

To assess model robustness and mitigate sampling bias, a k‐fold cross‐validation (*k* = 3) was performed on the training dataset, where the data was randomly split into three subsets of equal distribution to ensure diverse representation across each fold. Within each fold, GPA and PCA were fit using only the fold‐specific training data. To inform on the SSM validation, the following metrics were calculated: compactness, generalization, and specificity, in a similar manner mentioned above. Fold‐wise results were compared with evaluate stability of shape modes (PCs).

### 2.2 Anatomical Measurements

Since quantitative anatomical measures have previously correlated with the presence of severe lumbar spinal stenosis [3, 16, 17, 18, 19, 20] and qualitatively used to indicate stenosis severity and inform on spinal decompression surgery, our SSM results were used to evaluate the benefits of SSMs over other quantitative anatomical measures (facet angles, spinous process length, IVD diameters, facet width, and thecal sac diameter). Coronal and sagittal facet angles were measured between a line bisecting the facet joint (identified by two anatomical landmarks per joint) and either the coronal or sagittal reference plane, respectively. The spinous process length was measured between the spinous process tip and vertebral arch. The IVD diameters were measured between the lateral edges of the IVD (ML diameter) and between the anterior and posterior edges of the IVD (AP diameter). The facet width, while not a commonly used spinal measurement, was calculated as the linear distance of both the left and right articular surfaces. The thecal sac diameter was measured between the vertebral arch and the posterior edge of the IVD.

### 2.3 Statistical Analysis

To better characterize the MRI features underlying the stenosis classification, we performed chi‐square and Wilcoxon rank‐sum tests to assess the relationship between the stenosis groups and the assigned stenosis grade.

Differences between severe and normal stenosis MRIs were statistically assessed. Age and sex differences between groups were evaluated using a chi‐square test for sex and a linear model for age. The data was evaluated for normality and homoscedasticity using the Shapiro‐Wilks and Levene's tests. For normally distributed and homoscedasticity data (*p* > 0.05), a linear model that incorporated age as a covariate (due to potential differences between groups) was run followed by a Tukey post hoc analysis. When the data violated these assumptions (*p* < 0.05), the Kruskal‐Wallis with a Mann–Whitney U rank post hoc analysis was run. All statistical analyses were conducted using R version 4.5.0 [31], including the car package [32] for Levene's test and stats package [31] for ANOVA and Kruskal‐Wallis testing; with a significance of *p* < 0.05.

To evaluate whether traditional anatomical measurements and PC (shape modes) z‐scores could differentiate between severe and normal stenosis groups, receiver operating characteristics (ROC) curves were analyzed using 2000 stratified bootstrap replicates. The bootstrapped approach evaluates how reliable the threshold values are by repeatedly resampling the dataset. PCs with meaningful differences between groups, as well as all anatomical measurements, were used as predictive features. The severe stenosis group was treated as the positive class. An area under the curve (AUC) of 1.0 denotes a perfect classification, while an AUC of 0.5 suggests performance no better than random chance. In these plots, sensitivity (*y*‐axis) represents the true positive rate for severe stenosis, while 1—specificity (*x*‐axis) reflects the false positive rate. The optimal threshold was determined using Youden's criterion [33]. ROC curves and AUC were calculated in R version 4.5.0 [31] using the pROC and plotROC packages [34, 35].

In addition to the ROC analyses, a supervised classification approach was used to evaluate whether combining multiple PCs into one prediction could improve classification performance. A 10‐fold generalized linear model (GLM) with a binomial link function was implemented in R using the caret package. The model was trained using PCs that contributed to at least 5% of shape variance as predictors and severe stenosis (yes/no) as the binary outcome. Model performance was assessed using mean classification accuracy and Cohen's κ (kappa) to account for agreement beyond chance. The cross‐validation results were compared with ROC‐derived accuracy from PC1 (initial dataset only) to determine whether multivariate classification improved predictive performance.

To provide anatomical context to the PCs output from the SSM, PCs were correlated against anatomical measures using a multiway linear model. The PCs that were statistically different between severe and normal stenosis groups served as dependent variables, while anatomical measures, AP IVD diameter, ML IVD diameter, thecal sac diameter, facet joint orientation (coronal and sagittal angles), facet width, and posterior spinous process length, were considered as independent predictors. To avoid multicollinearity, only one parameter associated with highly correlated measures (e.g., ML and AP IVD diameters, the coronal and sagittal facet joint angles) were included, left and right facet widths were averaged, for the final analysis. Interactions between anatomical measures were also considered but shown to have minimal impact on the model's performance.

## 3 Results

### 3.1 Sample Composition

Our MRI dataset contained 140 MRIs from patients 50 years old or older. After removing blurry images and images with significant comorbidities, 41 images were left in the normal stenosis group and 21 images in the severe stenosis group. To keep the size of the normal and severe training datasets equal, 21 images from the 41 original normal stenosis images were randomly selected to create the normal stenosis training group and the remaining 20 images were used in the normal stenosis test group. Patient age and biological sex ratio (Female: Male) for each group (severe stenosis, normal stenosis training, and normal stenosis testing) were 64 ± 10, 56 ± 6, and 55 ± 6 years old and 10:11, 8:13, and 8:12, respectively. Age differed significantly between groups (Table S1). Both Lee and Schizas scores were significantly higher in the severe compared with the normal stenosis group (*p* < 0.01), supporting that the labeling surgeon focused on radiographic evidence of dural sac compression and neural crowding.

### 3.2 Reliability and SSM Validation

Inter‐ and intra‐rater reliability of the 13 anatomical landmarks demonstrated excellent agreement with the intra‐rater and inter‐rater ICCs above 0.94 (Table S2). The RMS Euclidean distance between repeated landmark placements was < 2.50 pixels (on average).

**FIGURE 2:**
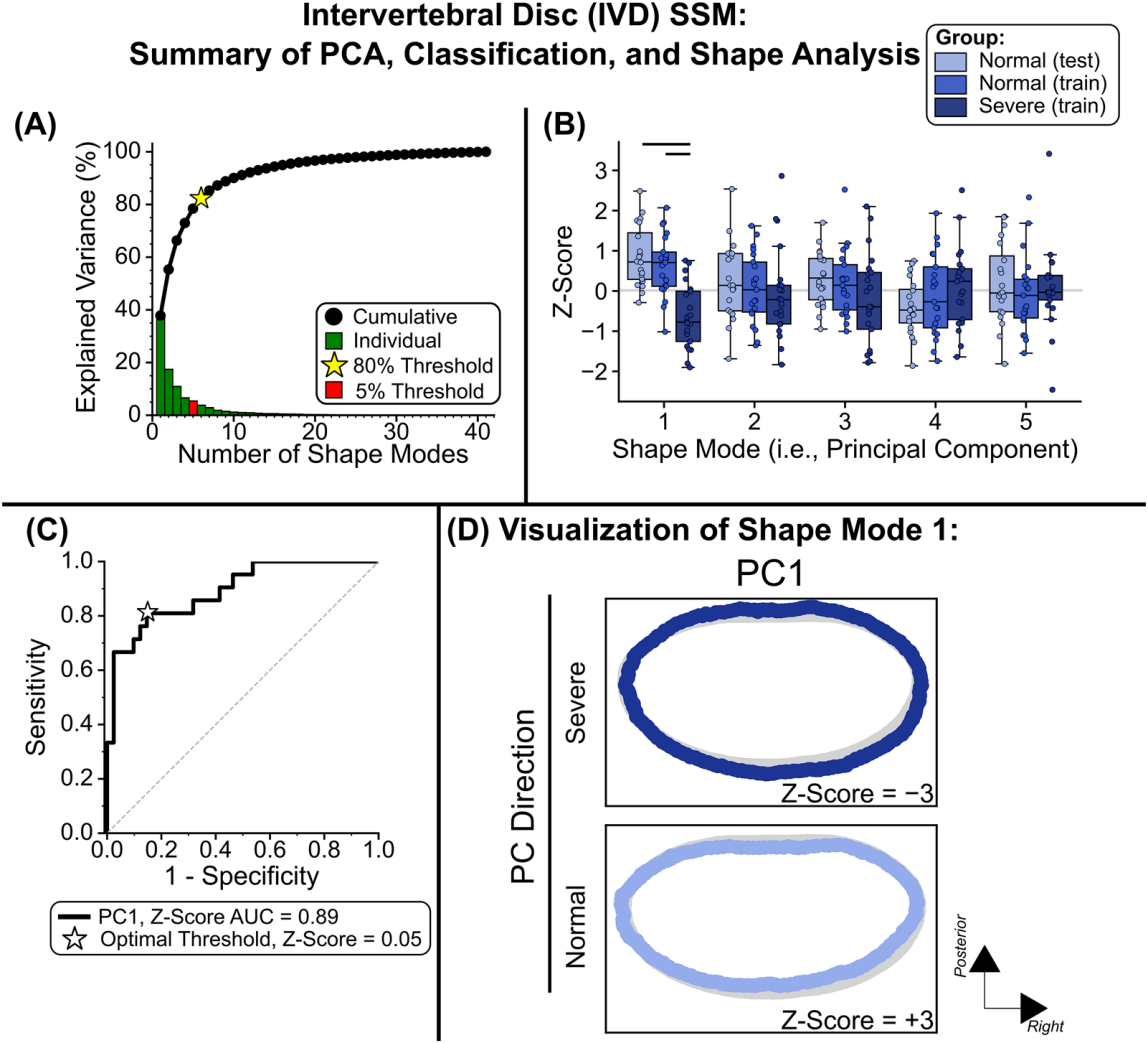
Intervertebral disc (IVD) statistical shape model (SSM) shows distinct differences between severe and normal stenosis groups (*p* < 0.05). (A) Explained variance for each principal component (PC) for individual (green bars) and cumulative (black circles). PC6 (yellow star) and PC5 (red bar) indicate 80% cumulative and 5% individual explained variance. (B) Z‐score analysis indicates that the only shape mode 1 (PC1) was associated with distinct shape differences between severe and normal stenosis groups. (C) ROC curve for PC1 (black solid line) with optimal threshold index (white star) indicates adequate distinction between severe and normal stenosis IVD shapes can be determined. (D) Visual of shape mode 1 (PC1) with ±3 z‐scores away (extreme deviations to clearly evaluate the small variations) from the mean shape (gray), show shape differences between severe (top row, dark blue) and normal (bottom row, light blue) stenosis groups.

**FIGURE 3:**
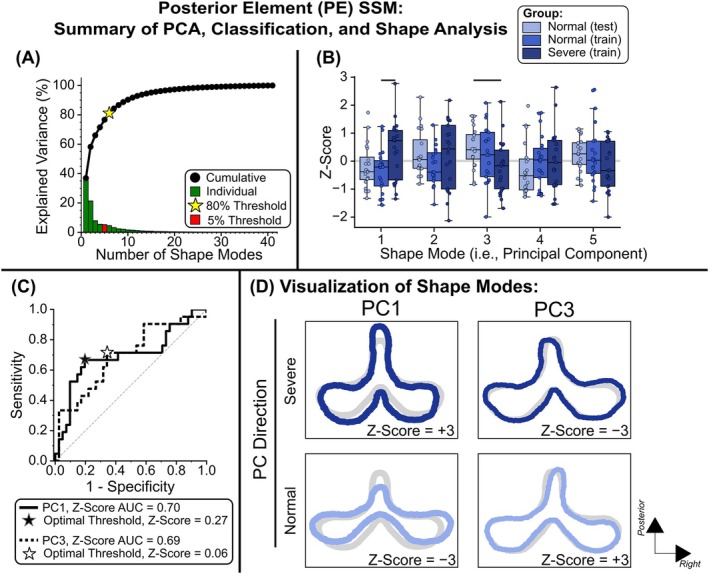
Posterior elements (PE) statistical shape model (SSM) shows differences between severe and normal stenosis groups (*p* < 0.05). (A) Explained variance for each principal component (PC) for individual (green bars) and cumulative (black circles). PC6 (yellow star) and PC5 (red bar) indicate 80% cumulative and 5% individual explained variance. (B) Z‐score analysis indicates that shape modes 1 and 3 (PC1, PC3) were associated with distinct shape differences between severe and normal stenosis groups. (C) ROC curve for PC1 (black solid line) & PC3 (black dashed line) with optimal threshold index (stars) indicates moderate ability to distinguish between severe and normal stenosis PE shapes. (D) Visual of shape modes (PC1, PC3) with ±3 z‐scores away (extreme deviations to clearly evaluate the small variations) from the mean shape (gray), show shape differences between severe (top row, dark blue) and normal (bottom row, light blue) stenosis groups.

**FIGURE 4:**
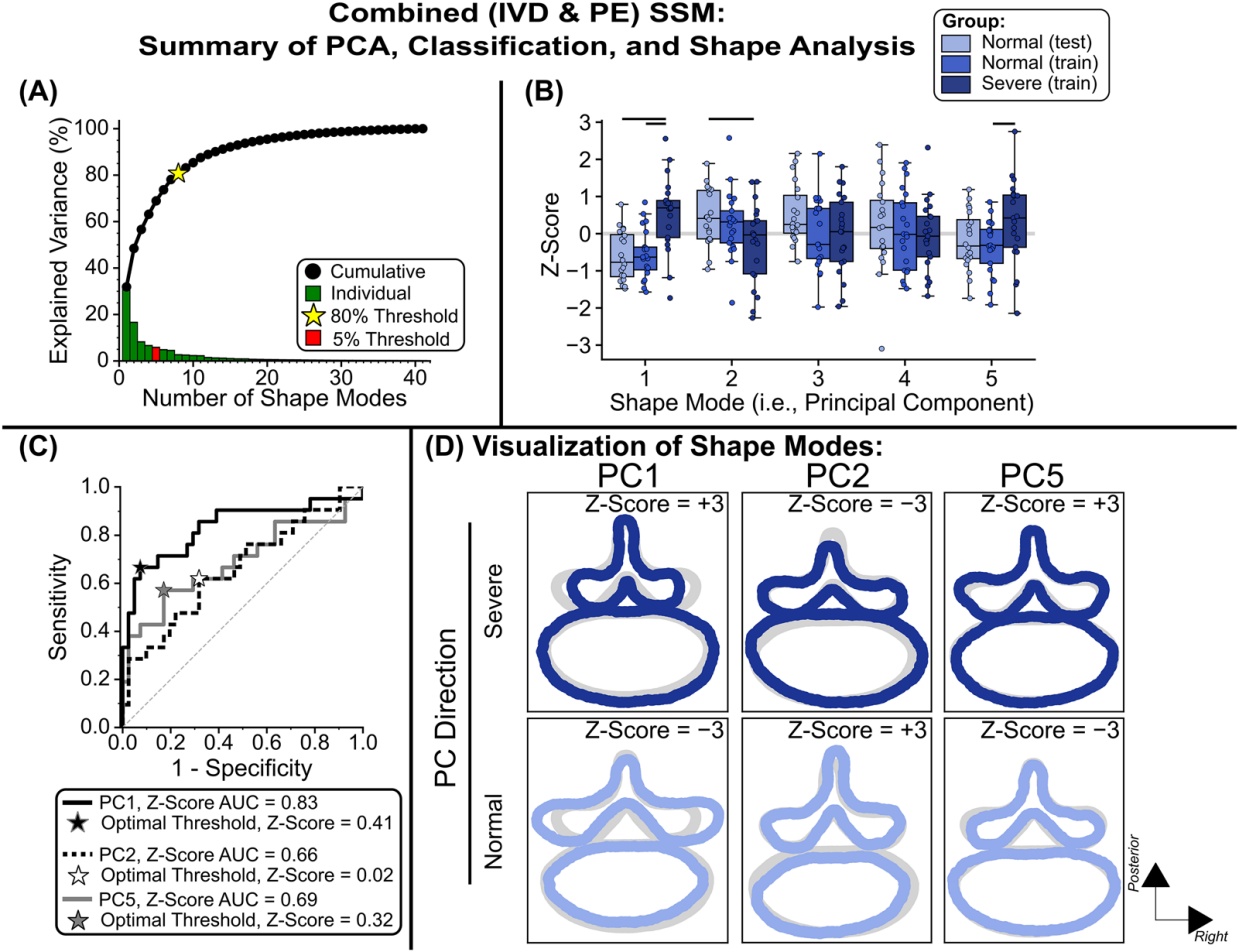
Combined (intervertebral disc—IVD & posterior element—PE) statistical shape model (SSM) shows distinct differences between severe and normal stenosis groups (*p* < 0.05). (A) Explained variance for each principal component (PC) for individual (green bars) and cumulative (black circles). PC8 (yellow star) and PC5 (red bar) indicate 80% cumulative and 5% individual explained variance. (B) Z‐score analysis indicates that shape modes 1, 2, and 5 (PC1, PC2, and PC5) were associated with distinct shape differences between severe and normal stenosis groups. (C) ROC curve for PC1 (black line), PC2 (black dashed line), and PC5 (gray solid line) with optimal threshold index (star) indicates strong and moderate ability to distinguish between severe and normal stenosis combined shapes. (D) Visual of shape modes (PC1, PC2, PC5) with ±3 z‐scores away (extreme deviations to clearly evaluate the small variations) from the mean shape (gray), show shape differences between severe (top row, dark blue) and normal (bottom row, light blue) stenosis groups.

The SSM validation analysis revealed consistent performance across the IVD, PE, and combined SSMs. Compactness curves indicated that the majority of shape variances were captured by the first 5 PCs, with diminishing gains from higher‐order PCs (Figures 2A, 3A, 4A; Figures S1A, S2A, S3A). Generalization error remained stable across k‐fold cross‐validation (Figures S1B, S2B, S3B), suggesting a robust PCA space not overly sensitive to the training data set. When evaluated on the test data, generalization error increased modestly with higher‐order PCs (Figure S4A), indicating that these PCs captured more data‐specific variation. Across the learned PCA space, the specificity error was consistent across the first 5 PCs (Figure S4B); however, specificity increased progressively with higher‐order PCs across the k‐fold cross‐validation (Figures S1C, S2C, S3C). Together, the specificity error reflected reduced anatomical plausibility of unconstrained randomly generated shapes as higher‐order PCs were added. Across all three SSMs, generalization and specificity errors exceeded inter‐patient anatomical variability (Table S3), indicating that the SSMs prioritize population‐level shape trends over high‐fidelity reconstruction of individual anatomies. Despite this, compactness curves demonstrated efficient representation of dominant shape variation, and low‐order PCs show stable and interpretable separation between normal and severe stenosis groups.

### 3.3 Intervertebral Disc (IVD) SSM

For the IVD SSM, 5 PCs were required to explain 78% of the total shape variance (compactness), where PC1 explained 38% (38.32% ± 1.36% across folds; Figure S1A) of the variance (Figure 2A). Only PC1 demonstrated a clear directional difference in z‐scores between groups (Figure 2B). Patients in the severe stenosis group exhibited PC1 z‐scores (−0.63 ± 0.80) in the opposite direction of the shape spectrum from the normal stenosis group (0.74 ± 0.76; *p* < 0.01). The test dataset (composed exclusively of normal stenosis patients) indicated that PC1 z‐scores were consistent between the training and test normal stenosis groups (0.63 ± 0.76 and 0.86 ± 0.76, *p* = 0.62). ROC analysis performed on the combined dataset demonstrated strong classification performance based on PC1 z‐scores (AUC 95% confidence interval [CI] = 0.81–0.98), with an optimal threshold CI of −0.53 to 0.70 (Table 1; Figure 2C). Visual inspection of PC1 deformations seems to show an increase in AP width in severe stenosis patients compared with normal stenosis patients (Figure 2D). Statistical analysis suggests that PC1 correlates with both the AP IVD (*p* < 0.01) and thecal sac diameters (*p* < 0.01, Table 2). Using the first 5 PCs from the IVD SSM, the GLM classifier achieved an accuracy of 0.85 ± 0.14, with a Cohen's κ of 0.64 ± 0.36, an accuracy similar to the results from the single‐PC‐based ROC (Table S4).

**TABLE 1:** ROC Analysis for Anatomical Measurements and Statistical Shape Model.

		AUC [95% CI]	Sensitivity	Specificity	Threshold [95% CI]
Raw Anatomical Measurements					
Thecal Sac	AP	0.84	0.81	0.78	14.39 mm
Spinous Process	AP	0.54	0.57	0.59	26.06 mm
IVD Diameter	AP	0.60	0.67	0.59	41.31 mm
	ML	0.60	0.33	0.93	60.86 mm
Facet Width	Left	0.69	0.71	0.63	18.61 mm
	Right	0.57	0.24	0.95	14.49 mm
	Both	0.63	0.62	0.61	18.59 mm
Coronal Facet Angle	Left	0.67	0.86	0.54	37.52°
	Right	0.61	0.29	0.95	55.96°
	Both	0.63	0.79	0.49	36.23°
Sagittal Facet Angle	Left	0.71	0.71	0.71	48.80°
	Right	0.53	0.24	0.95	34.85°
	Both	0.62	0.60	0.65	48.79°
Statistical Shape Models					
IVD	PC1	0.89 [0.81, 0.98]	0.81	0.85	0.05 [−0.53, 0.70]
PE	PC1	0.70 [0.54, 0.85]	0.67	0.8	0.27 [0.15, 0.71]
	PC3	0.69 [0.55, 0.84]	0.71	0.66	0.06 [−0.92, 0.67]
Combined	PC1	0.83 [0.71, 0.96]	0.67	0.93	0.41 [−0.50, 0.61]
	PC2	0.66 [0.51, 0.81]	0.62	0.68	0.02 [−1.04, 0.79]
	PC5	0.69 [0.53, 0.84]	0.57	0.83	0.32 [−0.50, 0.98]

*Note:* Highlights indicate thresholding ability: High threshold ability—AUC > 0.80 are dark green, medium‐high threshold ability—0.70 < AUC < 0.80 are medium green, medium—low threshold ability 0.66 < AUC < 0.70 are light green and low threshold ability AUC < 0.65 are white.

**TABLE 2:** Using a multiway linear model, correlations between the principal components (PCs) that showed differences due to severe stenosis and manual measures of the spine were created (* denoted *p* < 0.05, ** denotes *p* < 0.001).

Statistical shape model	PC	AP disc diameter (*p*)	Thecal sac diameter (*p*)	Coronal angle (*p*)	Facet width (*p*)	Spinous process length (*p*)	Overall Model Fit (*R* ^2^)
IVD	PC1	< 0.001**	< 0.001**	—	—	—	0.51**
PE	PC1	—	0.51	< 0.001**	0.05*	< 0.001**	0.66**
	PC3	—	< 0.05*	< 0.05*	0.26	0.08	0.29**
Combined	PC1	< 0.001**	0.16	< 0.001**	< 0.001**	< 0.001**	0.67**
	PC2	< 0.05*	< 0.001**	0.90	0.80	< 0.001**	0.53**
	PC5	0.41	< 0.05*	0.15	< 0.001**	0.14	0.27*

### 3.4 Posterior Element (PE) SSM

For the PE SSM, 5 PCs explained 77% of the total shape variance (compactness), where PC1 explained 37% (37.12% ± 5.74% across folds; Figure S2A) of the variance (Figure 3A). Two PCs showed differences between the groups, PC1 and PC3. Patients in the severe stenosis group exhibited positive PC1 z‐scores (0.39 ± 1.05), while those in the normal stenosis group had negative PC1 z‐scores (−0.39 ± 0.79; *p* < 0.05). Furthermore, PC1 z‐scores were consistent between the training and test normal stenosis groups (−0.39 ± 0.79 and −0.21 ± 0.79, *p* = 0.78). ROC analysis demonstrated moderate classification performance based on PC1 z‐scores (AUC 95% CI = 0.54–0.85), with an optimal threshold CI of 0.15 to 0.71 (Table 1; Figure 3C). Under visual examination, PC1 showed a decrease in the vertebral arch AP distance, and an increase in the spinous process length compared with the normal stenosis group (Figure 3D). Statistical analysis confirms these visual signs because PC1 correlates with the facet angles (*p* < 0.01) and the spinous process length (*p* < 0.01, Table 2). Using the first 5 PCs from the PE SSM, the GLM classifier achieved an accuracy of 0.66 ± 0.16 with a Cohen's κ of 0.13 ± 0.38, an accurate similar to the results from the single‐PC‐based ROC (Table S4).

PC3 explained 7.83% of the total shape variance. Z‐scores for the severe stenosis group (−0.24 ± 0.95) differed from the normal stenosis groups (test: 0.47 ± 0.72, *p* = 0.04; train: 0.24 ± 1.01, *p* = 0.20). PC3 z‐scores were consistent between the training and test normal stenosis groups (*p* = 0.70). ROC analysis demonstrated moderate classification performance based on PC3 z‐scores (AUC 95% CI = 0.55–0.84; Table 1). Under visual inspection, PC3 deformations revealed a slight decrease in the vertebral arch AP distance in severe compared with normal stenosis patients. Statistical analysis suggests that PC3 correlates with the thecal sac diameter (*p* < 0.05) and the facet angles (*p* < 0.05, Table 2).

### 3.5 Combined (IVD and PE) SSM

For the combined SSM, 5 PCs explained 69% of the total shape variance (compactness) where PC1 explained 32% (32.51% ± 4.43% across folds; Figure S3A) of the variance (Figure 4A). Three of the 5 PCs were different between the groups (PC1, PC2, and PC5). Patients in the severe stenosis group exhibited positive PC1 z‐scores (0.55 ± 0.99), while those in the normal stenosis group had negative PC1 z‐scores (−0.58 ± 0.66; *p* < 0.01). PC1 z‐scores were consistent between the training and test normal stenosis groups (−0.55 ± 0.66 and −0.61 ± 0.67, *p* = 0.97). ROC analysis demonstrated strong classification performance based on PC1 z‐scores (AUC 95% CI = 0.71–0.96), with an optimal threshold CI of −0.5 to 0.61 (Figure 4C). Visual inspection of combined SSM PC1 shows the severe stenosis group was associated with a narrowing of the thecal sac compared with the normal stenosis group (Figure 4D). Statistical analysis suggests that PC1 correlates with the AP IVD distance (*p* < 0.01), the facet angles (*p* < 0.01), the facet width (*p* < 0.01), and the spinous process length (*p* < 0.01, Table 2). Using the first 5 PCs from the combined SSM, the GLM classifier achieved an accuracy of 0.83 ± 0.16 with a Cohen's κ of 0.59 ± 0.38, an accurate similar to the single‐PC‐based ROC (Table S4).

PC2 explained 16.55% of the total shape variance. Z‐scores for the severe stenosis group (−0.26 ± 1.06) differed from the normal stenosis groups (test: 0.45 ± 0.76, *p* = 0.04; train: 0.26 ± 0.89, *p* = 0.17). PC2 z‐scores were consistent between the training and test normal stenosis groups (*p* = 0.79). ROC analysis demonstrated moderate to low classification performance based on PC2 z‐scores (AUC 95% CI = 0.51–0.81). Statistical analysis suggests that PC2 correlates with the AP IVD distance (*p* < 0.05), the thecal sac diameter (*p* < 0.01), and the spinous process length (*p* < 0.01, Table 2), some of which may explain the visual changes.

PC5 accounted for 5.77% of the total shape variance. Patients in the severe stenosis group had more positive PC5 z‐scores (0.34 ± 1.13) compared with the normal stenosis groups (test: −0.19 ± 0.79, *p* = 0.16; train: −0.34 ± 0.73, *p* < 0.05). PC5 z‐scores were consistent between the training and test normal stenosis groups (*p* = 0.87). ROC analysis demonstrated moderate to low classification performance based on PC5 z‐scores (AUC 95% CI = 0.53–0.84, Table 1). Visually, PC5 appears to correspond to a slight decrease in the thecal sac diameter for the severe stenosis group compared with the normal stenosis group, which is confirmed by multiway linear analysis of PC5 against the thecal sac diameter that suggests PC5 correlates with the thecal sac diameter (*p* < 0.05, Table 2) and facet width (*p* < 0.01, Table 2).

### 3.6 Traditional Anatomical Measurements

**FIGURE 5:**
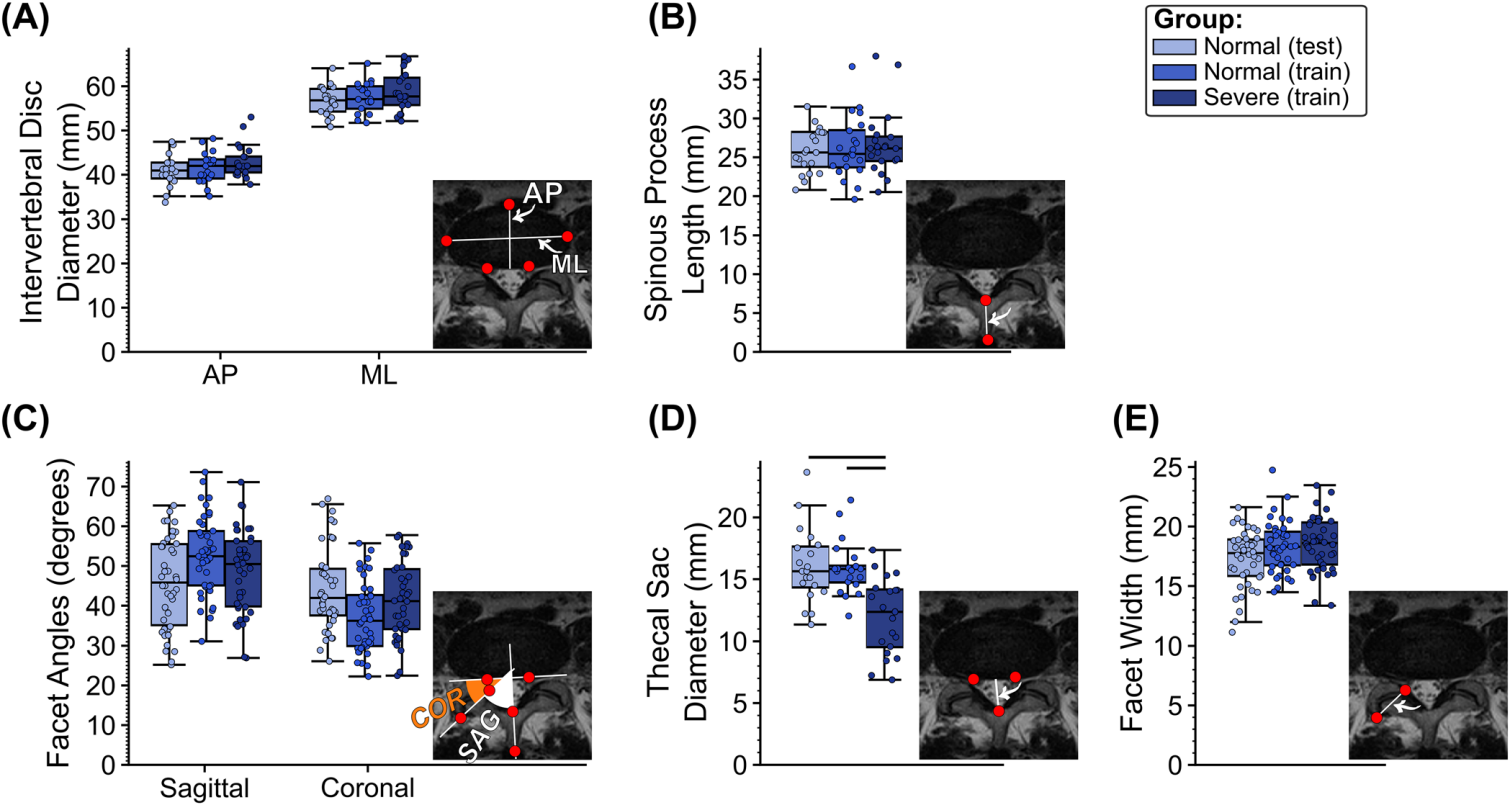
Manually measured anatomical measurements taken prior to image processing and shape alignment (A) intervertebral disc diameters, (B) spinous process length, (C) facet angles, (D) thecal sac diameter, and (E) facet width (*p* < 0.05).

To evaluate the benefits of SSMs over other quantitative spinal measures, raw anatomic measures were measured then compared with our SSM results. No differences were observed between the severe (58.83 ± 4.40 mm; 42.94 ± 3.77 mm) and normal (57.14 ± 3.33 mm; 41.22 ± 3.34 mm) stenosis patients for the ML and AP IVD diameters respectively (*p* = 0.32, *p* = 0.20; Figure 5A). No differences in the spinous process length or the facet angles were observed between severe and normal stenosis groups (Figure 5B,C). ROC analysis shows moderate to low thresholding potential, with sagittal left being the strongest (AUC = 0.71, Table 1). Trends towards statistical differences between groups based on the facet width were identified (*p* = 0.05, *p* = 0.07; Figure 5E). The manual thecal sac diameter measurement was much smaller for the severe stenosis (12.00 ± 3.05 mm) compared with the normal stenosis (15.98 ± 2.56 mm) patients (*p* < 0.01; Figure 5D). Thecal sac diameter had strong discriminatory power (AUC = 0.84); the optimal threshold was at 14.39 mm with a sensitivity of 0.81 and specificity of 0.78. The thecal sac diameter was the only anatomical measure with high ability to discriminate between groups, and this discriminatory power was similar to the combined SSM PC1's discriminatory power but below the IVD SSM PC1's discriminatory power (Table 1).

## 4 Discussion

In this study, a 2D SSM, derived from the central axial MRI slice at the L45 IVD level, distinguished between severe and normal stenosis patients within a low back pain population. By leveraging clinically routine 2D MRI rather than 3D imaging modalities, our approach aligns with current diagnostic workflows, supporting the feasibility of implementing SSMs in everyday clinical settings to expand knowledge about lumbar spinal stenosis diagnosis and treatment. Notably, while individual regions of interest, including the IVD and the PE, showed some ability to differentiate between groups, the combined SSM provided a more robust separation. This suggests that incorporating multiple anatomical features into a unified SSM offers a more comprehensive assessment of stenosis. Furthermore, the combined SSM outperformed several conventional quantitative anatomical measures, underscoring its potential to serve as a comprehensive tool for surgical planning and clinical decision‐making. In the analysis process, ROC curves of individual PCs and a supervised GLM classifier of multiple PCs were used, and both determined distinct shape difference between groups with comparable accuracy. This result aligns with previous shape modeling studies in musculoskeletal imaging, where the leading PC often captures the dominant clinically relevant variation [5, 10, 14, 15]. These findings also suggest that for these images, supervised classification using multiple PCs does not substantially outperform the first principal component, indicating that PC1 captures the majority of discriminative shape variance relevant to surgical indication.

To be effective, our 2D SSMs must capture known measures of severe stenosis. Accordingly, our IVD and combined SSMs were able to detect an increase in IVD diameter and a decrease in thecal sac diameter for patients with severe stenosis compared with those with normal stenosis. An increase in IVD diameters is expected because IVD impingement is a common comorbidity to severe (pain induced) stenosis and a clinically defined metric for performing decompression surgery [3, 16]. Additionally, the smaller thecal sac identified by the combined SSM as well as visual signs of reduced foraminal spacing are consistent with the presence of ligamentum flavum hypertrophy and reduced cerebrospinal fluid around the cauda equina, key indicators of severe lumbar spinal stenosis and the need for spinal decompression surgeries [4, 16, 17]. Together these results indicate that SSMs can detect features of thecal sac compression that may cause pain symptoms and indicate a benefit of spinal decompression surgery over non‐operative treatment.

Although the PE SSM suggested greater facet widening in severe stenosis patients, this did not correspond to statistically significant differences in manually measured facet angles or width. This apparent discrepancy reflects fundamental differences between what is captured by single linear measurements versus SSM. Facet angle primarily characterizes joint orientation relative to anatomical planes, whereas facet widening more closely reflects hypertrophic changes (such as bony overgrowth and capsular thickening) often associated with facet arthrosis and aging [3, 36]. Furthermore, facet hypertrophy has been identified as a main cause of nerve root compression in lumbar spinal stenosis [3, 36]. Despite the lack of significant group‐level differences in the manual measurements, statistically significant correlations were observed in multiway regression for PE SSM PC1 with facet angles and facet widths, as well as for PE SSM PC3 with thecal sac diameter and facet angles. These complex relationships indicate that the SSM captures subtle covariations between PE morphology and facet orientation that may be too small or spatially heterogeneous to yield significant differences when assessed using simple linear metrics.

Unexpectedly, the PE SSM also showed larger facets along with shorter posterior spinous processes in the normal aging spines compared with severely stenotic spines. This pattern is consistent with prior studies reporting that narrower facet widths may indicate sagittally aligned facets (larger coronal angle) observed in individuals diagnosed with severe stenosis or spondylolisthesis [20, 23, 37], compared with more coronally aligned facets (larger sagittal angles) that are associated with the general population. Similarly, previous work has shown that individuals diagnosed with severe stenosis tend to have greater posterior spinous process inclination angles [38], which could explain the increased posterior length visually observed captured by the SSMs. Together, these findings suggest that the PE SSM captures a combination of facet morphology, joint orientation, and PE geometry that reflects age‐related and degenerative remodeling processes, which may underlie biomechanical disruptions in patients with severe lumbar spinal stenosis [39]. In particular, changes in posterior spinous process geometry or facet orientation may affect the loading arm of spinal ligaments, thereby altering patient‐specific biomechanics and potentially contributing to the development of severe stenosis. Overall, the SSM‐based analysis of the PE reveals novel shape characteristics that could aid not only in improving surgical planning but also in advancing our understanding of the mechanisms underlying lumbar stenosis progression.

Our findings demonstrate that 2D SSMs from routine axial MRI slices can achieve similar or superior discriminatory performance in differentiating between severe and normal stenosis patients compared with current traditional anatomical measures. While earlier 3D models have provided comprehensive anatomical representations that show differences between patients based on age, sex, and posture, these 3D SSMs often require specialized imaging protocols and segmentation workflows not typically used in clinical practice [9, 11]. In contrast, our 2D MRI approach aligns with current imaging standards, enhancing clinical feasibility without sacrificing diagnostic utility. Axial MRI slices, in particular, were chosen for this analysis due to their ability to capture both central and foraminal stenosis. Although sagittal MRI slices might enhance this diagnosis [40], these images provide less accuracy on foraminal compromise, a main feature of foraminal stenosis [41]. Future work will focus on integrating axial and sagittal clinical MRI scans to construct a hybrid shape model that better represents the complex spatial characteristics of lumbar spinal stenosis. Furthermore, previous 2D SSMs have primarily focused on characterizing general shape variation or associating morphology with IVD degeneration, rather than linking vertebral body and IVD shapes directly to treatment decisions [12, 13, 14, 15]. Caution is warranted when interpreting specific AUC threshold values. The wide bootstrap intervals for the threshold value suggest that a single numeric threshold is unlikely to be stable without larger or external datasets. Future work will focus on deriving population‐invariant thresholds or using probabilistic classification. Nevertheless, this study demonstrated that 2D SSMs not only reflect morphological differences but also correlate with the presence of severe lumbar stenosis, which extends the clinical potential of SSMs.

The results from this study suggest that the SSM has strong potential to generalize well to unseen data while distinguishing between different anatomical shape categories. The consistent performance across the 3‐folds of cross‐validation highlights the model's robust predictive capabilities. Importantly, the generalization errors indicate that the model is particularly adept at classifying known shape categories.

This study has several limitations. First, severe stenosis labels were derived from imaging features alone and not validated against clinical symptoms, surgical recommendations, or postoperative outcomes. While this introduces subjectivity, our goal was to evaluate whether SSM‐derived shape patterns could distinguish morphologies consistent with severe lumbar stenosis (that may warrant further consideration for surgical evaluation) to assist with clinical diagnosis and surgical planning on MRI alone. Future work should extend this framework by incorporating multi‐observer grading and validating against surgical decision‐making and post‐operative outcomes. Second, only a single axial slice at the L45 level was analyzed, without incorporating multilevel or sagittal imaging data. This approach was intentional to test the feasibility of 2D SSMs in capturing localized morphological changes associated with severe stenosis from standard clinical imaging. Future work will extend this framework to multi‐slice or 3D models to evaluate shape variation along the full lumbar region. Third, the use of retrospective data in which ages differed between groups contributed to some observed effects. To account for this, all statistical models included age as a covariate, and future work will incorporate prospective, age‐ and sex‐matched cohorts to reduce confounding results. Lastly, although the SSM was validated via 3‐fold cross‐validation and the classification/ROC analyses used PC z‐scores from all patients, the fact that the independent test fold contained only normal patients is a limitation. This limitation paired with the elevated generalization and specificity errors suggest that the SSMs may still struggle with certain anatomical variations and degrees of stenosis, leading to potential misclassifications and overfitting in edge cases. Future work should focus on expanding the dataset, incorporating alternative validation metrics, and exploring advanced modeling techniques to enhance the robustness and clinical applicability of the SSM.

As with most clinical imaging studies, limitations surrounding the source and acquisition parameters exist. In this study, the source MRI dataset was drawn from a single institution, and scanner or protocol metadata were not reliable due to the de‐identification. Consequently, scanner‐related bias and harmonization of acquisition parameters across patients couldn't be formally assessed. However, the dataset included differences in number of echoes, repetition time, echo time, slice thickness, spacing between slices, imaging frequency, and number of phase encoding steps [27]. Second, the SegNet‐TL80 segmentation model was trained on a specific lumbar spine MRI dataset, which may limit generalizability to MRIs acquired with different scanners or protocols [27]. Despite these potential limitations, this dataset has been widely used and validated in several prior image analysis studies, suggesting its general suitability for morphological modeling and suitability for this initial proof of concept 2D SSM analysis [27, 42, 43, 44]. Future studies should incorporate multi‐institutional datasets and consider retraining (harmonization techniques) or fine‐tuning on a larger dataset to support broader clinical application and mitigate protocol‐related bias in shape mode estimation.

This study demonstrates that SSMs can be effectively implemented using standard 2D MRIs, offering a practical and accessible approach for clinical settings. Our findings indicate that SSMs perform as well as, or better than, existing quantitative clinical metrics in identifying patients with severe stenosis. Importantly, this method requires no changes to current imaging protocols, highlighting its ease of integration into routine clinical workflows. Future work will focus on applying these SSMs to datasets with known treatment outcomes to further validate their predictive power and to explore the treatment of other spinal disorders. This line of inquiry may ultimately enhance preoperative planning and support more personalized treatment strategies for patients with low back pain.

## Author Contributions

M.H.F., A.S., A.J., and J.M.M. contributed to the conception of the work and to the analysis and interpretation of the data. M.H.F. developed the model, additional codes, draft, and figures/tables. A.J. and W.M.D. performed manual grading assessments and clinical interpretations. All authors contributed to the editing of the manuscript.

## Funding

This work was supported by National Institutes of Health, T32 AR067708‐07.

## Conflicts of Interest

The authors declare no conflicts of interest.

## Supporting information


**Figure S1:** Intervertebral Disc (IVD) Statistical Shape Model (SSM) 3‐Fold Cross‐Validation performed exclusively within the training dataset and by iteratively holding‐out one‐fold for testing, following generalized Procrustes analysis. Individual folds are shown in blue, orange, and green (circles), and the mean across folds is shown in black (diamond). All reconstruction error metrics are reported as root‐mean‐squared (RMS) Euclidean distance normalized by the number of landmarks to facilitate comparison across models. (A) Compactness, expressed as cumulative explained variance, demonstrating the proportion of population shape variability captured as a function of PC modes. (B) Generalization, calculated as the reconstruction error between aligned test shapes and their PCA‐reconstructions; lower values indicate better generalization. (C) Specificity, calculated as the reconstruction error between 500 randomly generated shapes sampled from the PCA model and the closest real aligned shapes; lower value indicate more anatomically plausible shape generation. Across fold, compactness, generalization, and specificity curves exhibited consistent trends, indicating stable SSM behavior.
**Figure S2:** Posterior Element (PE) Statistical Shape Model (SSM) 3‐Fold Cross‐Validation performed exclusively within the training dataset and by iteratively holding‐out one‐fold for testing, following generalized Procrustes analysis. Individual folds are shown in blue, orange, and green (circles), and the mean across folds is shown in black (diamond). All reconstruction error metrics are reported as root‐mean‐squared (RMS) Euclidean distance normalized by the number of landmarks to facilitate comparison across models. (A) Compactness, expressed as cumulative explained variance, demonstrating the proportion of population shape variability captured as a function of PC modes. (B) Generalization, calculated as the reconstruction error between aligned test shapes and their PCA‐reconstructions; lower values indicate better generalization. (C) Specificity, calculated as the reconstruction error between 500 randomly generated shapes sampled from the PCA model and the closest real aligned shapes; lower value indicate more anatomically plausible shape generation. Across fold, compactness, generalization, and specificity curves exhibited consistent trends, indicating stable SSM behavior.
**Figure S3:** Combined (Intervertebral Disc & Posterior Elements) Statistical Shape Model (SSM) 3‐Fold Cross‐Validation performed exclusively within the training dataset and by iteratively holding‐out one‐fold for testing, following generalized Procrustes analysis. Individual folds are shown in blue, orange, and green (circles), and the mean across folds is shown in black (diamond). All reconstruction error metrics are reported as root‐mean‐squared (RMS) Euclidean distance normalized by the number of landmarks to facilitate comparison across models. (A) Compactness, expressed as cumulative explained variance, demonstrating the proportion of population shape variability captured as a function of PC modes. (B) Generalization, calculated as the reconstruction error between aligned test shapes and their PCA‐reconstructions; lower values indicate better generalization. (C) Specificity, calculated as the reconstruction error between 500 randomly generated shapes sampled from the PCA model and the closest real aligned shapes; lower value indicate more anatomically plausible shape generation. Across fold, compactness, generalization, and specificity curves exhibited consistent trends, indicating stable SSM behavior.
**Figure S4:** Statistical shape model (SSM) validation across the first 5 principal component (PC) modes for each model: Combined (blue), intervertebral disc (IVD, orange), and posterior element (PE, green), evaluated on the full dataset (*N* = 62). All reconstruction error metrics are reported as mean ± standard deviation of the root‐mean‐squared (RMS) Euclidean distance normalized by the number of landmarks to facilitate comparison across models. (A) Generalization, calculated as the reconstruction error between aligned test shape and their PCA‐reconstructions; lower values indicate improved generalization to unseen shapes. (B) Specificity, calculated as the reconstruction error between 500 randomly generated shapes sampled from the PCA model and the closest real aligned shape; lower values indicate greater anatomical plausibility of generated shapes. Across all three SSMs, generalization error decreased modestly as more PCs were included, while specificity error increased slightly, with overlapping standard deviations across PC modes, indicating stable model behavior.
**Table S1:** Demographic characteristics of the study cohort are stratified by group (severe or normal stenosis). Age is reported as mean ± standard deviation (SD), and biological sex is reported as counts. A significant difference in age was observed between the severe and the normal stenosis groups, while no difference in biological sex distribution was detected.
**Table S2:**. Intra‐rater and inter‐rater reliability of manually identified anatomical landmarks used to establish point correspondence for statistical shape modeling. For each anatomical region and landmark ID, the mean ± standard deviation (SD) Euclidean distance (in pixels) between repeated landmark placements is reported for intra‐rater (same rater, repeated sessions) and inter‐rater (between raters). Intraclass correlation coefficients (ICCs) are reported for both intra‐rater (ICC_intra_) and inter‐rater (ICC_inter_) reliability. Across all landmarks, intra‐rater mean distances ranged from 1.40 to 2.64 pixels and inter‐rater mean distances ranged from 1.14 to 3.94 pixels. ICCs values demonstrated excellent reliability for both intra‐rater (0.96–0.99) and inter‐rater (0.94–1.00) assessments, indicating consistent landmark placement and minimal observer‐dependent bias in the shape model pipeline.
**Table S3:** Quantitative summary of statistical shape model (SSM) validation metrics across the first 5 principal component (PC) modes for each model (intervertebral disc [IVD], posterior element [PE], and combined). Generalization and specificity are unitless and reported as mean ± standard deviation of the root‐mean‐squared (RMS) Euclidean distance between shapes, normalized by the number of landmarks. Inter‐patient variability represents the mean RMS distance between aligned shapes across patients within each SSM and is reported once per model. Across all SSMs and the first 5 PCs, inter‐patient variability was smaller than both generalization and specificity errors, and generalization error was consistently smaller than specificity error. These relationships indicate stable model behavior and support the use of low‐order PCs for population‐level shape analysis rather than high‐fidelity individual shape reconstruction.
**Table S4:** Comparison of classification performance using a single principal component (PC)‐based ROC threshold versus a supervised generalized linear model (GLM) classifier to determine severe or normal stenosis. ROC‐based accuracy was computed using a threshold on PC1 *z*‐scores derived from each statistical shape model (SSM; intervertebral disc [IVD], posterior element [PE], & combined [IVD + PE]). GLM classification used the first 5 PCs as input features and was evaluated using k‐fold cross‐validation, with accuracy and Cohen's kappa (κ) reported as mean ± standard deviation.

## Data Availability

The data that support the findings of this study are available from the corresponding author upon reasonable request.

## References

[1] J. N. Katz , Z. E. Zimmerman , H. Mass , and M. C. Makhni , “Diagnosis and Management of Lumbar Spinal Stenosis: A Review,” Journal of the American Medical Association 327, no. 17 (2022): 1688–1699. 35503342 10.1001/jama.2022.5921

[2] A. J. Weiss , A. Elixhauser , and R. M. Andrews , “Characteristics of Operating Room Procedures in U.S. Hospitals, 2011,” in Healthcare Cost and Utilization Project (HCUP) Statistical Briefs (Agency for Healthcare Research and Quality, 2006). 24716251

[3] E. Siebert , H. Pruss , R. Klingebiel , V. Failli , K. M. Einhaupl , and J. M. Schwab , “Lumbar Spinal Stenosis: Syndrome, Diagnostics and Treatment,” Nature Reviews. Neurology 5, no. 7 (2009): 392–403. 19578346 10.1038/nrneurol.2009.90

[4] C. Schizas , N. Theumann , A. Burn , et al., “Qualitative Grading of Severity of Lumbar Spinal Stenosis Based on the Morphology of the Dural Sac on Magnetic Resonance Images,” Spine (Phila Pa 1976) 35, no. 21 (2010): 1919–1924. 20671589 10.1097/BRS.0b013e3181d359bd

[5] N. Sarkalkan , H. Weinans , and A. A. Zadpoor , “Statistical Shape and Appearance Models of Bones,” Bone 60 (2014): 129–140. 24334169 10.1016/j.bone.2013.12.006

[6] S. Alimohamadi Gilakjan , H. Majedi , B. Makki Abadi , and A. Ahmadian , “Spinal Pain Relief Procedures With the Assistance of the MRI‐Updated Statistical Shape Model,” International Journal of Medical Robotics 16, no. 3 (2020): e2085. 10.1002/rcs.2085 31995264

[7] A. L. Clouthier , J. Wenghofer , E. K. Wai , and R. B. Graham , “Morphable Models of the Lumbar Spine to Vary Geometry Based on Pathology, Demographics, and Anatomical Measurements,” Journal of Biomechanics 146 (2023): 111421. 36603365 10.1016/j.jbiomech.2022.111421

[8] J. Q. Campbell and A. J. Petrella , “Automated Finite Element Modeling of the Lumbar Spine: Using a Statistical Shape Model to Generate a Virtual Population of Models,” Journal of Biomechanics 49, no. 13 (2016): 2593–2599. 27270207 10.1016/j.jbiomech.2016.05.013

[9] J. F. M. Hollenbeck , C. M. Cain , J. A. Fattor , P. J. Rullkoetter , and P. J. Laz , “Statistical Shape Modeling Characterizes Three‐Dimensional Shape and Alignment Variability in the Lumbar Spine,” Journal of Biomechanics 69 (2018): 146–155. 29402403 10.1016/j.jbiomech.2018.01.020

[10] J. R. Meakin , J. S. Gregory , F. W. Smith , F. J. Gilbert , and R. M. Aspden , “Characterizing the Shape of the Lumbar Spine Using an Active Shape Model: Reliability and Precision of the Method,” Spine (Phila Pa 1976) 33, no. 7 (2008): 807–813. 18379410 10.1097/BRS.0b013e31816949e6

[11] L. Tang , Z. Hu , Y. S. Lin , and J. Hu , “A Statistical Lumbar Spine Geometry Model Accounting for Variations by Age, Sex, Stature, and Body Mass Index,” Journal of Biomechanics 130 (2022): 110821. 34749159 10.1016/j.jbiomech.2021.110821

[12] A. H. Ali , A. B. Cowan , J. S. Gregory , R. M. Aspden , and J. R. Meakin , “The Accuracy of Active Shape Modelling and End‐Plate Measurements for Characterising the Shape of the Lumbar Spine in the Sagittal Plane,” Computer Methods in Biomechanics and Biomedical Engineering 15, no. 2 (2012): 167–172. 10.1080/10255842.2010.518962 22268530

[13] J. R. Meakin , J. S. Gregory , R. M. Aspden , F. W. Smith , and F. J. Gilbert , “The Intrinsic Shape of the Human Lumbar Spine in the Supine, Standing and Sitting Postures: Characterization Using an Active Shape Model,” Journal of Anatomy 215, no. 2 (2009): 206–211. 19493187 10.1111/j.1469-7580.2009.01102.x PMC2740968

[14] F. Rieger , D. A. Rothenfluh , S. J. Ferguson , and D. Ignasiak , “Comprehensive Assessment of Global Spinal Sagittal Alignment and Related Normal Spinal Loads in a Healthy Population,” Journal of Biomechanics 170 (2024): 112127. 38781798 10.1016/j.jbiomech.2024.112127

[15] J. A. Deane , A. V. Pavlova , A. K. P. Lim , J. S. Gregory , R. M. Aspden , and A. H. McGregor , “Is Intrinsic Lumbar Spine Shape Associated With Lumbar Disc Degeneration? An Exploratory Study,” BMC Musculoskeletal Disorders 21, no. 1 (2020): 433. 32620099 10.1186/s12891-020-03346-7 PMC7334848

[16] G. Andreisek , R. A. Deyo , J. G. Jarvik , et al., “Consensus Conference on Core Radiological Parameters to Describe Lumbar Stenosis ‐ an Initiative for Structured Reporting,” European Radiology 24, no. 12 (2014): 3224–3232. 25079488 10.1007/s00330-014-3346-z

[17] M. Zileli , M. Crostelli , M. Grimaldi , et al., “Natural Course and Diagnosis of Lumbar Spinal Stenosis: WFNS Spine Committee Recommendations,” World Neurosurg X 7 (2020): 100073. 32613187 10.1016/j.wnsx.2020.100073 PMC7322797

[18] J. Abbas , N. Peled , I. Hershkovitz , and K. Hamoud , “Facet Tropism and Orientation: Risk Factors for Degenerative Lumbar Spinal Stenosis,” BioMed Research International 2020 (2020): 2453503. 32685454 10.1155/2020/2453503 PMC7341411

[19] E. Akar and H. Somay , “Comparative Morphometric Analysis of Congenital and Acquired Lumbar Spinal Stenosis,” Journal of Clinical Neuroscience 68 (2019): 256–261. 31331753 10.1016/j.jocn.2019.07.015

[20] X. Liu , X. Zhao , Y. Long , et al., “Facet Sagittal Orientation: Possible Role in the Pathology of Degenerative Lumbar Spinal Stenosis,” Spine (Phila Pa 1976) 43, no. 14 (2018): 955–958. 29189570 10.1097/BRS.0000000000002493

[21] M. H. Foltz , A. H. Seidenstein , C. Almeida , A. Kim , A. Jain , and J. M. Middendorf , “A Quantitative Review of Finite Element‐Based Biomechanics of Lumbar Decompression Surgery,” Biomechanics and Modeling in Mechanobiology 24, no. 3 (2025): 743–759, 10.1007/s10237-025-01936-9. 40392425

[22] D. Degulmadi , B. R. Dave , and A. Krishnan , “Age‐ and Sex‐Related Changes in Facet Orientation and Tropism in Lower Lumbar Spine: An MRI Study of 600 Patients,” European Spine Journal 28, no. 5 (2019): 961–966. 30887218 10.1007/s00586-019-05953-y

[23] Y. Masharawi , B. Rothschild , K. Salame , G. Dar , S. Peleg , and I. Hershkovitz , “Facet Tropism and Interfacet Shape in the Thoracolumbar Vertebrae: Characterization and Biomechanical Interpretation,” Spine (Phila Pa 1976) 30, no. 11 (2005): E281–E292. 15928537 10.1097/01.brs.0000164098.00201.8d

[24] P. Oura , J. Karppinen , J. Niinimaki , and J. A. Junno , “Sex Estimation From Dimensions of the Fourth Lumbar Vertebra in Northern Finns of 20, 30, and 46 Years of Age,” Forensic Science International 290 (2018): 350.e1–350.e6. 10.1016/j.forsciint.2018.07.011 30078665

[25] M. Travis Caton, Jr. , W. F. Wiggins , S. R. Pomerantz , and K. P. Andriole , “Effects of Age and Sex on the Distribution and Symmetry of Lumbar Spinal and Neural Foraminal Stenosis: A Natural Language Processing Analysis of 43,255 Lumbar MRI Reports,” Neuroradiology 63, no. 6 (2021): 959–966. 33594502 10.1007/s00234-021-02670-6 PMC8128837

[26] S. Sudirman , Y. Widyaningsih , S. M. M. Al Arif , and K. Ramli , “Lumbar Spine MRI Dataset,” In. V2 ed. Mendeley Data, 2019.

[27] A. S. Al‐Kafri , S. Sudirman , A. Hussain , et al., “Boundary Delineation of MRI Images for Lumbar Spinal Stenosis Detection Through Semantic Segmentation Using Deep Neural Networks,” IEEE Access 7 (2019): 43487–43501.

[28] M. Matsumoto and Y. Kurita , “Twisted GFSR Generators,” ACM Transactions on Modeling and Computer Simulation 2 (1992): 179–194.

[29] J. Gower , “Generalized Procrustes Analysis,” Psychometrika 40 (1975): 33–51.

[30] “Principal Component Analysis for Special Types of Data,” in Principal Component Analysis (Springer, 2002).

[31] R Core Team , R: A Language and Environment for Statistical Computing [Computer Program] (R Foundation for Statistical Computing, 2025).

[32] J. Fox and S. Weisberg , An R Companion to Applied Regression, Third ed. (Sage, 2019).

[33] W. J. Youden , “Index for Rating Diagnostic Tests,” Cancer 3, no. 1 (1950): 32–35. 15405679 10.1002/1097-0142(1950)3:1<32::aid-cncr2820030106>3.0.co;2-3

[34] X. Robin , N. Turck , A. Hainard , et al., “pROC: An Open‐Source Package for R and S+ to Analyze and Compare ROC Curves,” BMC Bioinformatics 12 (2011): 77. 21414208 10.1186/1471-2105-12-77 PMC3068975

[35] M. S. Sachs , “plotROC: A Tool for Plotting ROC Curves,” Journal of Statistical Software, Code Snippets 79 (2017): 1–19. 10.18637/jss.v079.c02 PMC6347406 30686944

[36] A. Saifuddin , “The Imaging of Lumbar Spinal Stenosis,” Clinical Radiology 55, no. 8 (2000): 581–594. 10964728 10.1053/crad.2000.0223

[37] J. Grogan , B. H. Nowicki , T. A. Schmidt , and V. M. Haughton , “Lumbar Facet Joint Tropism Does Not Accelerate Degeneration of the Facet Joints,” AJNR. American Journal of Neuroradiology 18, no. 7 (1997): 1325–1329. 9282864 PMC8338045

[38] J. Abbas , N. Peled , I. Hershkovitz , and K. Hamoud , “Spinous Process Inclination in Degenerative Lumbar Spinal Stenosis Individuals,” BioMed Research International 2020 (2020): 8875217. 33381595 10.1155/2020/8875217 PMC7755483

[39] C. E. Aylott , R. Puna , P. A. Robertson , and C. Walker , “Spinous Process Morphology: The Effect of Ageing Through Adulthood on Spinous Process Size and Relationship to Sagittal Alignment,” European Spine Journal 21, no. 5 (2012): 1007–1012. 21959943 10.1007/s00586-011-2029-6 PMC3337914

[40] E. R. Glenn , A. H. Seidenstein , C. H. Savage , et al., “Artificial Intelligence for Lumbar Spine Anatomy and Pathology Detection: A Scoping Review,” Journal of Orthopaedic Reports (2025): 100760, 10.1016/j.jorep.2025.100760.

[41] W. Kim , K. S. Ahn , C. H. Kang , W. Y. Kang , and K. S. Yang , “Comparison of MRI Grading for Cervical Neural Foraminal Stenosis Based on Axial and Oblique Sagittal Images: Concordance and Reliability Study,” Clinical Imaging 43 (2017): 165–169. 28334616 10.1016/j.clinimag.2017.03.008

[42] J. T. Silveira , S. Girisha , and P. P. Kundapur , “Automated Lumbar Spine Segmentation in MRI Using an Enhanced U‐Net With Inception Module and Dual‐Output Mechanism,” Scientific Reports 15, no. 1 (2025): 39215. 41214055 10.1038/s41598-025-20721-3 PMC12603128

[43] G. Ghobrial and C. Roth , “Deep Learning‐Based Automated Segmentation and Quantification of the Dural Sac Cross‐Sectional Area in Lumbar Spine MRI,” Frontiers in Radiology 5 (2025): 1503625. 40201339 10.3389/fradi.2025.1503625 PMC11975661

[44] T. Shahzadi , M. U. Ali , F. Majeed , et al., “Nerve Root Compression Analysis to Find Lumbar Spine Stenosis on MRI Using CNN,” Diagnostics (Basel) 13, no. 18 (2023): 2975. 37761342 10.3390/diagnostics13182975 PMC10529899

