# Supplementary material for: Application of Statistical Shape Models to Standard Lumbar MRI for Stenosis Treatment Stratification: Severe Versus Normal Stenosis
Source: JOR Spine. 2026 Jun 7;9(2):e70189. doi: 10.1002/jsp2.70189 (PMC13243780; doi:10.1002/jsp2.70189)
Supplement: Supplementary file 1 — Figure S1: Intervertebral Disc (IVD) Statistical Shape Model (SSM) 3‐Fold Cross‐Validation performed exclusively within the training dataset and by iteratively holding‐out one‐fold for testing, following generalized Procrustes analysis. Individual folds are shown in blue, orange, and green (circles), and the mean across folds is shown in black (diamond). All reconstruction error metrics are reported as root‐mean‐squared (RMS) Euclidean distance normalized by the number of landmarks to facilitate comparison across models. (A) Compactness, expressed as cumulative explained variance, demonstrating the proportion of population shape variability captured as a function of PC modes. (B) Generalization, calculated as the reconstruction error between aligned test shapes and their PCA‐reconstructions; lower values indicate better generalization. (C) Specificity, calculated as the reconstruction error between 500 randomly generated shapes sampled from the PCA model and the closest real aligned shapes; lower value indicate more anatomically plausible shape generation. Across fold, compactness, generalization, and specificity curves exhibited consistent trends, indicating stable SSM behavior. Figure S2: Posterior Element (PE) Statistical Shape Model (SSM) 3‐Fold Cross‐Validation performed exclusively within the training dataset and by iteratively holding‐out one‐fold for testing, following generalized Procrustes analysis. Individual folds are shown in blue, orange, and green (circles), and the mean across folds is shown in black (diamond). All reconstruction error metrics are reported as root‐mean‐squared (RMS) Euclidean distance normalized by the number of landmarks to facilitate comparison across models. (A) Compactness, expressed as cumulative explained variance, demonstrating the proportion of population shape variability captured as a function of PC modes. (B) Generalization, calculated as the reconstruction error between aligned test shapes and their PCA‐reconstructions; l [file JSP2-9-e70189-s001.docx]

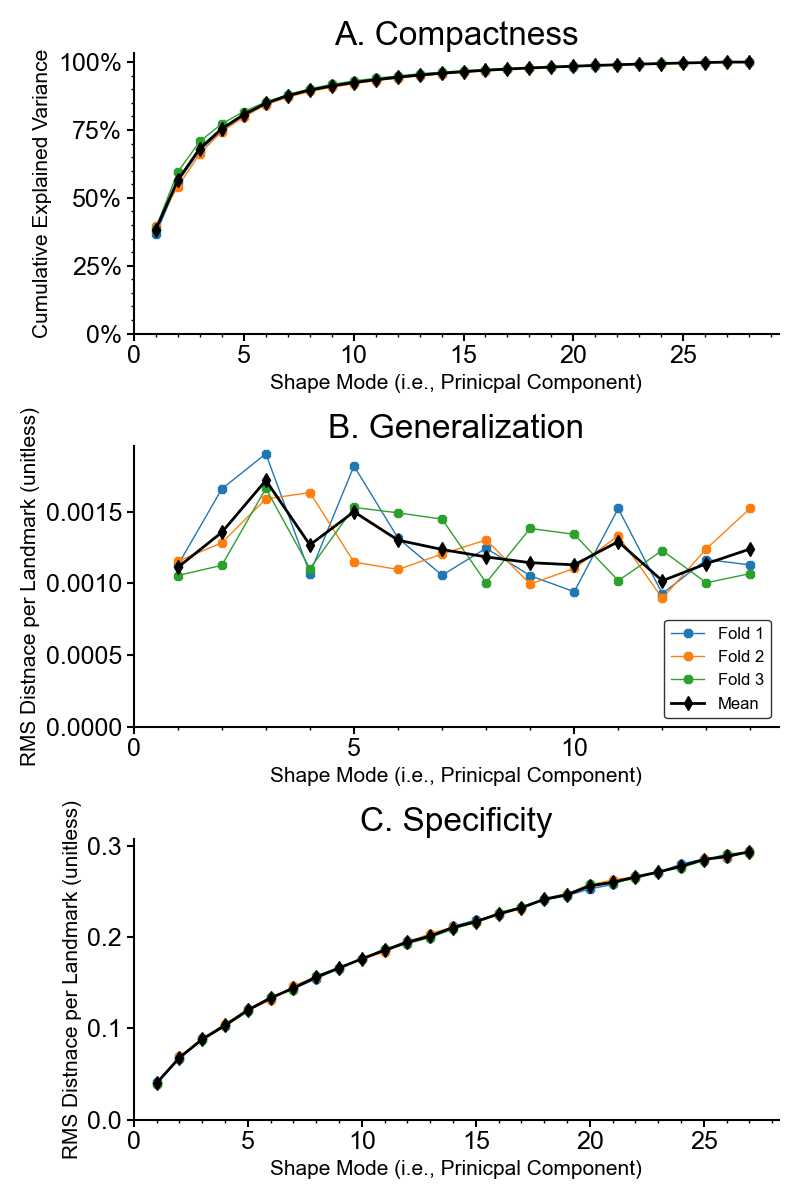


Supplemental Figure S1. Intervertebral Disc (IVD) Statistical Shape Model (SSM) 3-Fold Cross-Validation performed exclusively within the training dataset and by iteratively holding-out one-fold for testing, following generalized Procrustes analysis. Individual folds are shown in blue, orange, and green (circles), and the mean across folds is shown in black (diamond). All reconstruction error metrics are reported as root-mean-squared (RMS) Euclidean distance normalized by the number of landmarks to facilitate comparison across models. (A) Compactness, expressed as cumulative explained variance, demonstrating the proportion of` population shape variability captured as a function of PC modes. (B) Generalization, calculated as the reconstruction error between aligned test shapes and their PCA-reconstructions; lower values indicate better generalization. (C) Specificity, calculated as the reconstruction error between 500 randomly generated shapes sampled from the PCA model and the closest real aligned shapes; lower value indicate more anatomically plausible shape generation. Across fold, compactness, generalization, and specificity curves exhibited consistent trends, indicating stable SSM behavior.


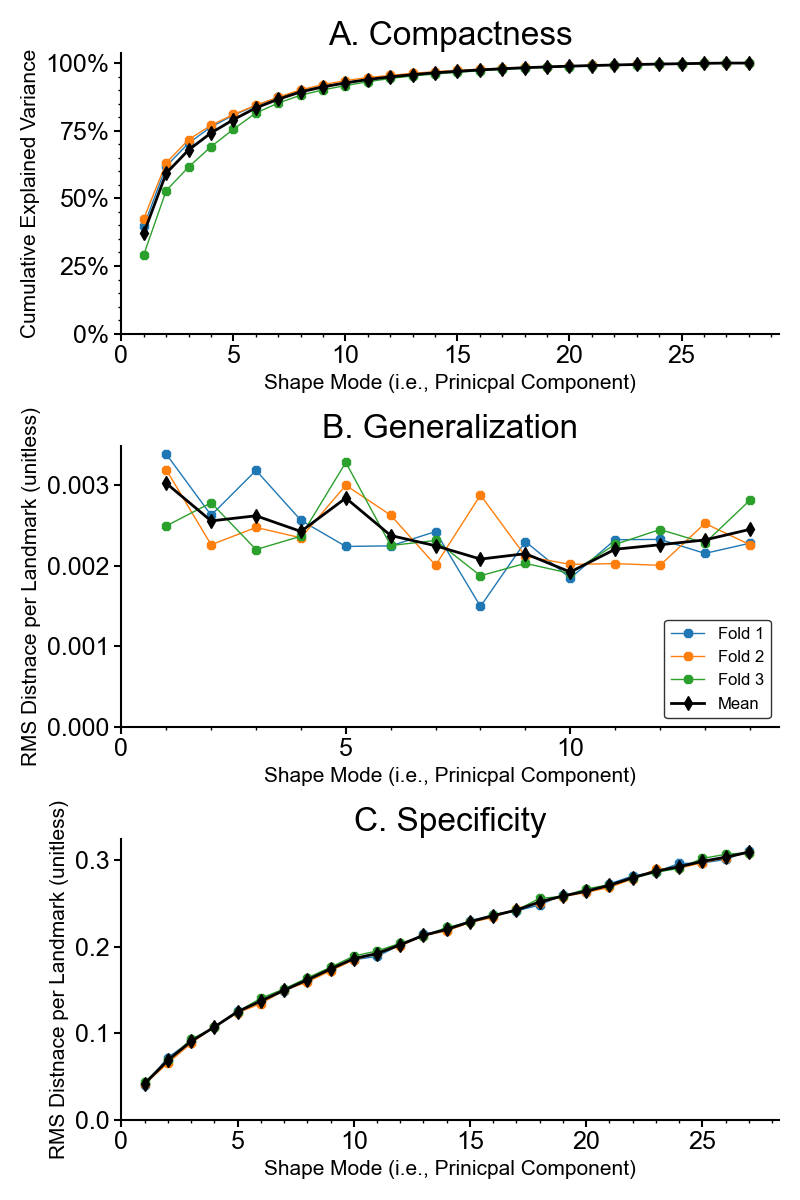


Supplemental Figure S2. Posterior Element (PE) Statistical Shape Model (SSM) 3-Fold Cross-Validation performed exclusively within the training dataset and by iteratively holding-out one-fold for testing, following generalized Procrustes analysis. Individual folds are shown in blue, orange, and green (circles), and the mean across folds is shown in black (diamond). All reconstruction error metrics are reported as root-mean-squared (RMS) Euclidean distance normalized by the number of landmarks to facilitate comparison across models. (A) Compactness, expressed as cumulative explained variance, demonstrating the proportion of` population shape variability captured as a function of PC modes. (B) Generalization, calculated as the reconstruction error between aligned test shapes and their PCA-reconstructions; lower values indicate better generalization. (C) Specificity, calculated as the reconstruction error between 500 randomly generated shapes sampled from the PCA model and the closest real aligned shapes; lower value indicate more anatomically plausible shape generation. Across fold, compactness, generalization, and specificity curves exhibited consistent trends, indicating stable SSM behavior.


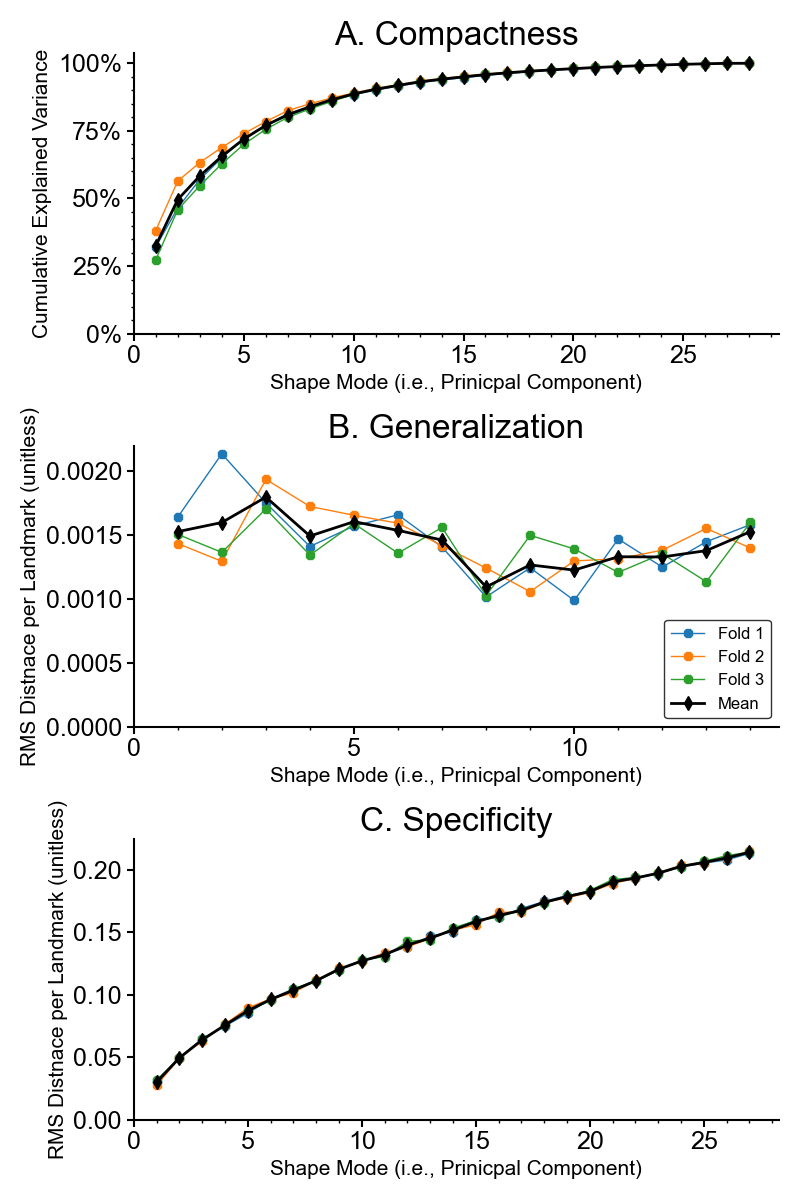


Supplemental Figure S3. Combined (Intervertebral Disc & Posterior Elements) Statistical Shape Model (SSM) 3-Fold Cross-Validation performed exclusively within the training dataset and by iteratively holding-out one-fold for testing, following generalized Procrustes analysis. Individual folds are shown in blue, orange, and green (circles), and the mean across folds is shown in black (diamond). All reconstruction error metrics are reported as root-mean-squared (RMS) Euclidean distance normalized by the number of landmarks to facilitate comparison across models. (A) Compactness, expressed as cumulative explained variance, demonstrating the proportion of` population shape variability captured as a function of PC modes. (B) Generalization, calculated as the reconstruction error between aligned test shapes and their PCA-reconstructions; lower values indicate better generalization. (C) Specificity, calculated as the reconstruction error between 500 randomly generated shapes sampled from the PCA model and the closest real aligned shapes; lower value indicate more anatomically plausible shape generation. Across fold, compactness, generalization, and specificity curves exhibited consistent trends, indicating stable SSM behavior.


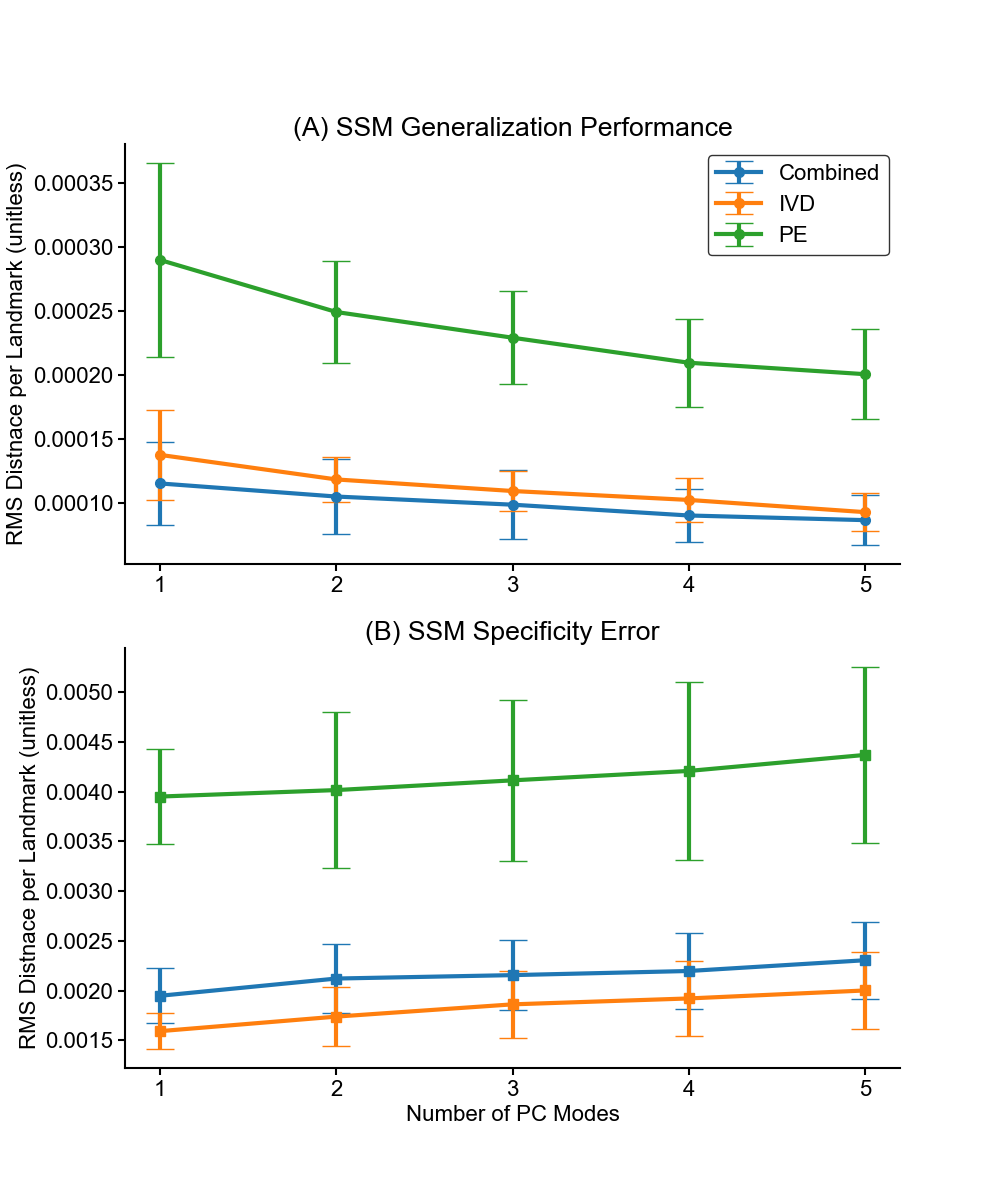


Supplemental Figure S4. Statistical shape model (SSM) validation across the first 5 principal component (PC) modes for each model: Combined (blue), intervertebral disc (IVD, orange), and posterior element (PE, green), evaluated on the full dataset (N=62). All reconstruction error metrics are reported as mean ± standard deviation of the root-mean-squared (RMS) Euclidean distance normalized by the number of landmarks to facilitate comparison across models. (A) Generalization, calculated as the reconstruction error between aligned test shape and their PCA-reconstructions; lower values indicate improved generalization to unseen shapes. (B) Specificity, calculated as the reconstruction error between 500 randomly generated shapes sampled from the PCA model and the closest real aligned shape; lower values indicate greater anatomical plausibility of generated shapes. Across all three SSMs, generalization error decreased modestly as more PCs were included, while specificity error increased slightly, with overlapping standard deviations across PC modes, indicating stable model behavior.

Supplemental Table S1. Demographic characteristics of the study cohort are stratified by group (severe or normal stenosis). Age is reported as mean ± standard deviation (SD), and biological sex is reported as counts. A significant difference in age was observed between the severe and the normal stenosis groups, while no difference in biological sex distribution was detected.

|  | Severe Stenosis | Normal Stenosis | p-value |
| --- | --- | --- | --- |
| Age (years) | 63.1±10.5 | 55.7±6.1 | <0.001 |
| Sex (M/F) | 11/10 | 25/16 | 0.71 |

Supplemental Table S2. Intra-rater and inter-rater reliability of manually identified anatomical landmarks used to establish point correspondence for statistical shape modeling. For each anatomical region and landmark ID, the mean ± standard deviation (SD) Euclidean distance (in pixels) between repeated landmark placements is reported for intra-rater (same rater, repeated sessions) and inter-rater (between raters). Intraclass correlation coefficients (ICCs) are reported for both intra-rater (ICC_intra_) and inter-rater (ICC_inter_) reliability. Across all landmarks, intra-rater mean distances ranged from 1.40-2.64 pixels and inter-rater mean distances ranged from 1.14-3.94 pixels. ICCs values demonstrated excellent reliability for both intra-rater (0.96-0.99) and inter-rater (0.94-1.00) assessments, indicating consistent landmark placement and minimal observer-dependent bias in the shape model pipeline.

| Region | Landmark ID | Intra Distance (Mean ± SD) | Inter Distance (Mean ± SD) | ICC_intra_ | ICC_inter_ |
| --- | --- | --- | --- | --- | --- |
| IVD | Left | 2.63 ± 2.42 | 2.21 ± 1.82 | 0.96 | 0.98 |
|  | Anterior | 2.64 ± 2.05 | 2.78 ± 1.95 | 0.98 | 0.98 |
|  | Right | 2.33 ± 1.82 | 3.50 ± 2.57 | 0.98 | 0.98 |
|  | Posterior Right | 2.46 ± 1.89 | 3.94 ± 2.80 | 0.99 | 0.97 |
|  | Posterior Left | 2.55 ± 1.87 | 2.46 ± 1.86 | 0.98 | 0.98 |
| PE | Spinous Process | 1.67 ± 1.00 | 1.14 ± 0.89 | 0.99 | 1.00 |
|  | Lamina Left | 2.58 ± 1.97 | 2.98 ± 2.14 | 0.98 | 0.97 |
|  | Transverse Process Left Posterior | 2.39 ± 1.74 | 3.28 ± 2.41 | 0.98 | 0.97 |
|  | Transverse Process Left Anterior | 2.17 ± 1.96 | 2.26 ± 1.67 | 0.98 | 0.98 |
|  | Vertebral Arch | 1.40 ± 1.49 | 1.40 ± 2.83 | 0.99 | 0.98 |
|  | Transverse Process Right Anterior | 1.45 ± 1.28 | 3.03 ± 4.12 | 0.99 | 0.94 |
|  | Transverse Process Right Posterior | 2.21 ± 1.59 | 2.67 ± 3.09 | 0.99 | 0.98 |
|  | Lamina Right | 2.43 ± 1.67 | 3.81 ± 2.58 | 0.98 | 0.95 |

Supplemental Table S3. Quantitative summary of statistical shape model (SSM) validation metrics across the first 5 principal component (PC) modes for each model (intervertebral disc [IVD], posterior element [PE], and combined). Generalization and specificity are unitless and reported as mean ± standard deviation of the root-mean-squared (RMS) Euclidean distance between shapes, normalized by the number of landmarks. Inter-patient variability represents the mean RMS distance between aligned shapes across patients within each SSM and is reported once per model. Across all SSMs and the first 5 PCs, inter-patient variability was smaller than both generalization and specificity errors, and generalization error was consistently smaller than specificity error. These relationships indicate stable model behavior and support the use of low-order PCs for population-level shape analysis rather than high-fidelity individual shape reconstruction.

| Statistical Shape Model | Principal Component | Generalization | Specificity | Inter-Patient Variability |
| --- | --- | --- | --- | --- |
| IVD | PC1 | (13.77 ± 3.51) x 10^-5^ | (15.92 ± 1.79) x 10^-4^ | 5.15 x 10^-6^ |
|  | PC2 | (11.85 ± 1.75) x 10^-5^ | (17.38 ± 2.97) x 10^-4^ |  |
|  | PC3 | (10.95 ± 1.57) x 10^-5^ | (18.61 ± 3.35) x 10^-4^ |  |
|  | PC4 | (10.25 ± 1.72) x 10^-5^ | (19.21 ± 3.81) x 10^-4^ |  |
|  | PC5 | (9.30 ± 1.51) x 10^-5^ | (20.01 ± 3.83) x 10^-4^ |  |
| PE | PC1 | (29.01 ± 7.58) x 10^-5^ | (39.51 ± 4.80) x 10^-4^ | 23.21 x 10^-6^ |
|  | PC2 | (24.94 ± 3.97) x 10^-5^ | (40.16 ± 7.85) x 10^-4^ |  |
|  | PC3 | (22.94 ± 3.63) x 10^-5^ | (41.13 ± 8.07) x 10^-4^ |  |
|  | PC4 | (20.98 ± 3.43) x 10^-5^ | (42.08 ± 8.91) x 10^-4^ |  |
|  | PC5 | (20.08 ± 3.52) x 10^-5^ | (43.69 ± 8.83) x 10^-4^ |  |
| Combined | PC1 | (11.54 ± 3.24) x 10^-5^ | (19.48 ± 2.74) x 10^-4^ | 5.48 x 10^-6^ |
|  | PC2 | (10.51 ± 2.92) x 10^-5^ | (21.21 ± 3.46) x 10^-4^ |  |
|  | PC3 | (9.88 ± 2.67) x 10^-5^ | (21.55 ± 3.48) x 10^-4^ |  |
|  | PC4 | (9.03 ± 2.09) x 10^-5^ | (21.97 ± 3.86) x 10^-4^ |  |
|  | PC5 | (8.67 ± 1.94) x 10^-5^ | (23.05 ± 3.88) x 10^-4^ |  |

Supplemental Table S4. Comparison of classification performance using a single principal component (PC)-based ROC threshold versus a supervised generalized linear model (GLM) classifier to determine severe or normal stenosis. ROC-based accuracy was computed using a threshold on PC1 z-scores derived from each statistical shape model (SSM; intervertebral disc [IVD], posterior element [PE], & combined [IVD + PE]). GLM classification used the first 5 PCs as input features and was evaluated using k-fold cross-validation, with accuracy and Cohen’s kappa (κ) reported as mean ± standard deviation.

|  | ROC Model - PC1 | Classification GLM with k-fold cross-validation | |
| --- | --- | --- | --- |
| SSM Model | Accuracy | Accuracy | κ |
| IVD | 0.84 | 0.85 ± 0.14 | 0.64 ± 0.36 |
| PE | 0.76 | 0.66 ± 0.16 | 0.13 ± 0.38 |
| Combined | 0.84 | 0.83 ± 0.16 | 0.59 ± 0.38 |
